# African Hydroclimate During the Early Eocene From the DeepMIP Simulations

**DOI:** 10.1029/2022PA004419

**Published:** 2022-05-16

**Authors:** Charles J. R. Williams, Daniel J. Lunt, Ulrich Salzmann, Tammo Reichgelt, Gordon N. Inglis, David R. Greenwood, Wing‐Le Chan, Ayako Abe‐Ouchi, Yannick Donnadieu, David K. Hutchinson, Agatha M. de Boer, Jean‐Baptiste Ladant, Polina A. Morozova, Igor Niezgodzki, Gregor Knorr, Sebastian Steinig, Zhongshi Zhang, Jiang Zhu, Matthew Huber, Bette L. Otto‐Bliesner

**Affiliations:** ^1^ School of Geographical Sciences University of Bristol Bristol UK; ^2^ NCAS/Department of Meteorology University of Reading Reading UK; ^3^ Geography and Environmental Sciences Northumbria University Newcastle upon Tyne UK; ^4^ Department of Geosciences University of Connecticut Mansfield CT USA; ^5^ School of Ocean and Earth Science University of Southampton Southampton UK; ^6^ Department of Biology Brandon University Brandon MB Canada; ^7^ Atmosphere and Ocean Research Institute The University of Tokyo Tokyo Japan; ^8^ Centre Européen de Recherche et d’Enseignement des Géosciences de l'Environnement Aix‐en‐Provence France; ^9^ Department of Geological Sciences Stockholm University Stockholm Sweden; ^10^ Climate Change Research Centre University of New South Wales Sydney NSW Australia; ^11^ Laboratoire des Sciences du Climat et de l’Environnement Gif‐sur‐Yvette France; ^12^ Institute of Geography Russian Academy of Sciences Moscow Russia; ^13^ Institute of Geological Sciences Polish Academy of Sciences Warsaw Poland; ^14^ Alfred Wegener Institute for Polar and Marine Research Bremerhaven Germany; ^15^ Bjerknes Centre for Climate Research University of Bergen Bergen Norway; ^16^ Climate and Global Dynamics Laboratory National Center for Atmospheric Research Boulder CO USA; ^17^ Department of Earth, Atmospheric and Planetary Sciences Purdue University West Lafayette IN USA

**Keywords:** paleoclimate, DeepMIP, arly Eocene, African precipitation

## Abstract

The early Eocene (∼56–48 Myr ago) is characterized by high CO_2_ estimates (1,200–2,500 ppmv) and elevated global temperatures (∼10°C–16°C higher than modern). However, the response of the hydrological cycle during the early Eocene is poorly constrained, especially in regions with sparse data coverage (e.g., Africa). Here, we present a study of African hydroclimate during the early Eocene, as simulated by an ensemble of state‐of‐the‐art climate models in the Deep‐time Model Intercomparison Project (DeepMIP). A comparison between the DeepMIP pre‐industrial simulations and modern observations suggests that model biases are model‐ and geographically dependent, however, these biases are reduced in the model ensemble mean. A comparison between the Eocene simulations and the pre‐industrial suggests that there is no obvious wetting or drying trend as the CO_2_ increases. The results suggest that changes to the land sea mask (relative to modern) in the models may be responsible for the simulated increases in precipitation to the north of Eocene Africa. There is an increase in precipitation over equatorial and West Africa and associated drying over northern Africa as CO_2_ rises. There are also important dynamical changes, with evidence that anticyclonic low‐level circulation is replaced by increased south‐westerly flow at high CO_2_ levels. Lastly, a model‐data comparison using newly compiled quantitative climate estimates from paleobotanical proxy data suggests a marginally better fit with the reconstructions at lower levels of CO_2_.

## Key Points

- State‐of‐the‐art climate models are used to study African hydroclimate during the early Eocene (approximately 50 Myr ago)
- With increasing levels of CO_2_, there are changes to African precipitation, due to dynamical changes such as low‐level circulation
- A comparison between the models and newly compiled climate estimates shows a marginally better match at lower levels of CO_2_

## 1 Introduction

One of the ways to better understand future anthropogenic‐induced climate change is to simulate past climates, using these as partial analogs for the future and allowing the testing of climate models to simulate climates very different from today (Braconnot et al., 2011; Tierney et al., 2020). Simulating past climates allows not only an interrogation of the mechanisms of past climate change (Haywood et al., 2020; Lunt et al., 2021), but if a robust comparison with available proxy data can be produced, this allows confidence in future climate change projections that are often based on models tuned to a modern climate state (Harrison et al., 2014; Taylor et al., 2011; Williams et al., 2020, 2021; Zhu et al., 2020).

It has long been known that African precipitation, and in particular that over West Africa, is of vital importance to the more than one billion people in sub‐Saharan Africa who survive predominantly on rain‐fed agriculture and, concurrently, are highly vulnerable to extreme precipitation events causing both flooding and drought (Williams & Kniveton, 2011). However, a lack of weather and climate data across much of the continent has resulted in a high level of uncertainty concerning both present day and future climate trends (Salerno et al., 2019), and although it is expected that both average temperature and precipitation will increase across Africa along with the rest of the world (IPCC, 2021), regional variation is particularly high across Africa.

Due to their particular relevance to African precipitation, two Quaternary time periods have recently been investigated by Williams et al. (2020) under the Paleoclimate Modeling Intercomparison Project (PMIP, Braconnot et al., 2007), now in its 4th phase and itself under the umbrella of the Coupled Model Intercomparison Project, now in its 6th phase (CMIP6, Eyring et al., 2016). These time periods are the mid‐Holocene (6,000 yr ago, 6 ka) and Last Interglacial (127 ka). However, excess warmth and enhancement of the Northern Hemisphere during these periods is caused primarily by changes to the orbital configuration of Earth, rather than elevated greenhouse gases (Kageyama et al., 2018). To investigate substantial greenhouse gas‐induced warming, and its result on regional hydroclimate such as across Africa, periods further back in time are needed, and two such candidates in the context of PMIP are the mid‐Pliocene (∼3 Myr ago, 3 Ma) and the early Eocene (∼56.05–47.8 Ma). However, with CO_2_ levels ranging from 316 to 420 ppmv during the mid‐Pliocene (Martínez‐Botí et al., 2015), this is more similar to modern levels rather than being a suitable analog for future projections by the end of the 21st century; using the previous RCP 8.5 scenario, this could be over 1,000 ppmv (IPCC, 2013). The early Eocene, with CO_2_ levels ranging between 1,200 and 2,500 ppmv (Anagnostou et al., 2020, 2016; Lunt et al., 2021), is comparable to the current future projections, and in particular for the extended high‐emissions/low‐mitigation scenarios such as in the year 2,300 under SSP5‐8.5 (Arias et al., 2021). As a result of this high CO_2_, the early Eocene was a period characterized by temperatures up to ∼5°C higher than today in the tropics (e.g., Cramwinckel et al., 2018; Gaskell et al., 2022, Inglis et al., 2020; Pearson & Wade, 2007), and much greater polar amplification with temperatures reaching ∼20°C warmer than today at terrestrial high latitudes (e.g., Huber & Caballero, 2011; Naafs et al., 2018; van Dijk et al., 2020).

Despite being a partial analog for future climate change, until the last few years climate model simulations of high CO_2_ periods such as the early Eocene have not been evaluated within a consistent framework (Lunt et al., 2017); the closest to this was an informal model‐data comparison, considering four climate models, known as the Eocene Model Intercomparison Project (EoMIP), undertaken by Lunt et al. (2012). This work focused on temperature‐based metrics, however, another study by Carmichael et al. (2016) used the same EoMIP ensemble to look at the hydrological cycle and hydroclimate changes in response to the elevated CO_2_ levels in the early Eocene. The results focusing specifically on Africa are discussed in more detail below but, globally, when compared to proxy data it was found that the models generally underestimated precipitation over high latitudes, and those models showing the most warming in these regions gave the best match to the data (Carmichael et al., 2016). Concerning the impact of elevated CO_2_, it was found that all early Eocene simulations showed a more intense hydrological cycle (relative to the pre‐industrial era, hereafter PI), with enhanced global precipitation and evaporation, and that this was generally directly related to the elevated temperatures resulting from higher CO_2_ (Carmichael et al., 2016). At any given level of CO_2_, global precipitation changes varied widely between models, and certain regions (such as tropical Africa, discussed further below) were found to be sensitive to which model was assessed (Carmichael et al., 2016).

However, a disadvantage (albeit unavoidable) to EoMIP was that there was no consistent framework to the models' experimental design; each used different boundary conditions (e.g., paleogeography) and different levels of CO_2_ (Lunt et al., 2012). To resolve this problem, therefore, more recently the Deep Time Model Intercomparison Project (DeepMIP) was envisaged and conducted, using CMIP3 and CMIP5 models as well as some of the most recent state‐of‐the‐art CMIP6‐class models (Lunt et al., 2017). The large‐scale features coming out of the simulations are discussed in Lunt et al. (2021), with several conclusions being drawn. First, boundary conditions other than CO_2_, discussed in Section 2.1, contributed between 3°C and 5°C of the global mean early Eocene warming, relative to the PI (Lunt et al., 2021). Second, the DeepMIP simulations showed less of a temperature spread than the models in EoMIP, and an increase in climate sensitivity (Lunt et al., 2021). Lastly, when compared to proxy SST data, most models reproduced the large‐scale spatial patterns of the reconstructions but still struggled at the regional scale, such as in the south‐west Pacific (Lunt et al., 2021).

Similar to Lunt et al. (2012), Lunt et al. (2021) only focused on temperature and CO_2_‐based metrics. The majority of recent studies looking at Eocene hydroclimate have focused on reconstructing evidence for the Asian monsoon (e.g., Farnsworth et al., 2019; Licht et al., 2014; Ma et al., 2019; Quan et al., 2012; Xie et al., 2019). There are very few studies, and in particular modeling studies, focusing on Africa. The aforementioned study by Carmichael et al. (2016) using the EoMIP ensemble found that tropical Africa was particularly sensitive to the model in question, and that the models varied in skill (when reproducing precipitation, relative to observations) in regions of relatively low precipitation such as over northern Africa's Sahel region. Moreover, although some models showed similar PI precipitation over tropical Africa, under early Eocene conditions they were quite different (Carmichael et al., 2016). It should be noted, however, that this study did not actually include any early Eocene mean annual precipitation (MAP) reconstructions from Africa, only some Lutetian samples (∼41–47 Ma). More recently, Carmichael et al. (2018) ran several CO_2_ simulations using just the UK Met Office Hadley Centre model HadCM3L, finding an increase in both the size and frequency of extreme precipitation events over equatorial and East Africa. Although MAP changes were relatively small, extreme rainfall increased by up to 70% over parts of tropical Africa, with summer precipitation events dominating the regime over southern Africa (Carmichael et al., 2018). Another example of Eocene African work is that of X. D. Liu et al. (2019), who looked at the Asian, African and Australian monsoons across five different time periods and found that precipitation from the African monsoon existed as early as the mid‐Paleocene. Keery et al. (2018) found the variability of Asian and African precipitation during the Eocene was predominantly accounted for by orbital configuration changes such as the precession and obliquity; in DeepMIP, however, these were kept at PI values and so, here, the impact on African precipitation will only be down to the CO_2_ or the other boundary condition changes.

In this paper four main questions are addressed:

1. How well do the DeepMIP models' PI simulations reproduce modern observations of African precipitation?
2. What is the impact of CO_2_ and other early Eocene boundary conditions on African precipitation in the DeepMIP models' early Eocene simulations?
3. What are the physical mechanisms behind this precipitation response?
4. How do the DeepMIP models' early Eocene simulations compare with proxy data of African precipitation?

Section 2 of this article briefly describes the experimental design followed by the DeepMIP models, gives a brief introduction to the models themselves, and describes the observational and proxy data used for comparative purposes. Section 3 presents the results, addressing each of the above questions. Section 4 summarizes and concludes.

## 2 Experiment Design, Models, and Proxy Data

### 2.1 Experiment Design

The full experimental design, which all DeepMIP modeling groups were required to follow as closely as possible, is detailed extensively in Lunt et al. (2017) and so will only be briefly outlined here. In addition to the various CO_2_ experiments, all modeling groups were required to carry out a PI simulation for comparison purposes, which was to be as close as possible to the CMIP6 standard *piControl* simulation (Eyring et al., 2016).

**Figure 1:**
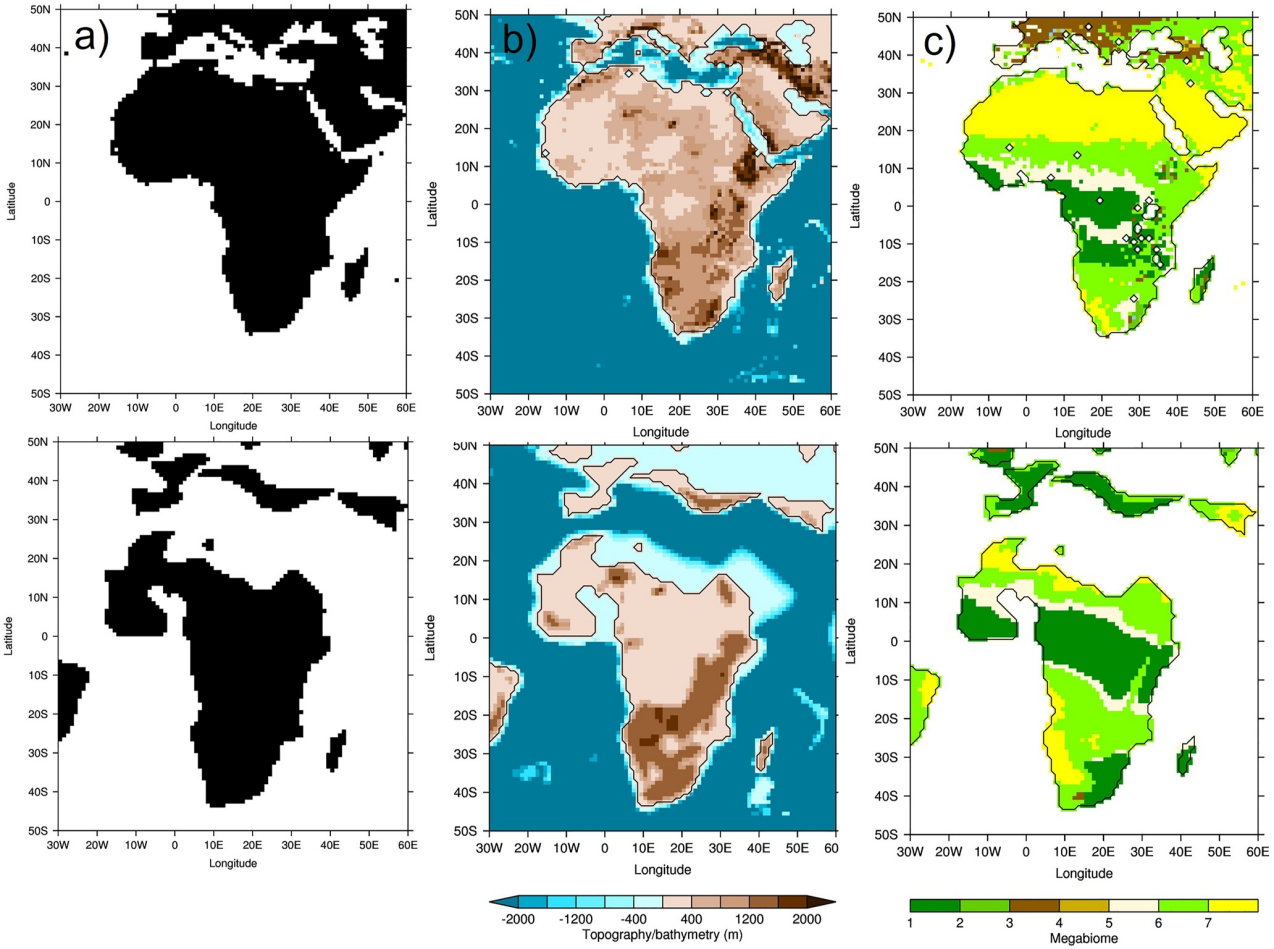
Main boundary conditions changed in Deep‐time Model Intercomparison Project (DeepMIP) simulations, where top row = PI and bottom row = early Eocene: (a) land sea mask; (b) topography/bathymetry; (c) vegetation, expressed as megabiomes according to Harrison and Prentice (2003) (where 1 = tropical, 2 = warm‐temperate, 3 = temperate, 4 = boreal, 5 = savanna, 6 = grassland and 7 = desert). The PI topography/bathymetry is taken from ETOPO5, re‐gridded to 1° × 1° resolution, whereas the other fields are from Herold et al. (2014).

For the early Eocene simulations, a number of boundary conditions needed to be changed, the key ones for the African region of which are shown in Figure 1.

First, the land sea mask (LSM) was based on the paleogeographic heights (discussed further below), with possible manual manipulation required in some models to maintain the various gateways (Lunt et al., 2017). The new LSM produced a geographically smaller Africa relative to the PI, with much of the present‐day landmass north of 20°N being ocean in the early Eocene due to the increased sea level (Figure 1a). Second, the paleogeography (including topography and bathymetry) was based on the digital reconstruction of the early Eocene from Herold et al. (2014), with the topography (and sub‐grid scale topography) being applied as an absolute value rather than as an anomaly (Lunt et al., 2017). Over Africa, the most pronounced changes were over southern and eastern Africa, with generally larger areas of raised topography in the early Eocene, relative to the PI (Figure 1b). This can be seen more clearly in the Supporting Information, where the differences in topography are shown; there is clearly a large increase in elevation over western Africa where there is land in the early Eocene but ocean in the PI, but apart from this (where the landmasses coincide) the largest changes are over southern and eastern Africa (Figure S1 in Supporting Information S1). Third, concerning the land surface, vegetation and river run‐off routing was also based on the data set of Herold et al. (2014), using an appropriate lookup table to convert the vegetation megabiomes into whatever format was required by the model (Lunt et al., 2017). The early Eocene vegetation was created by running the dynamic vegetation model BIOME4 (Kaplan et al., 2003), with the resulting 27 biomes being consolidated into 10 megabiomes following the procedure of Harrison and Prentice (2003); please see Table 3 in Harrison and Prentice (2003) for a distinction between these megabiomes. BIOME4 itself was forced by Eocene topography, bathymetry and CO_2_ coming out of an early Eocene simulation from the CESM climate model. Concerning how well the simulated vegetation compares with reconstructions, Herold et al. (2014) state that it compares well with vegetation inferred from Paleocene and Eocene palynoflora (Morley, 2007; Utescher & Mosbrugger, 2007), and is consistent with geological indicators of climate (Crowley, 2012). Although Herold et al. (2014) highlight a dry bias in vegetation over South America, there is no specific mention of Africa, primarily because there is currently little or no paleobotanical data for Africa, meaning validation was not possible. Although it is beyond the scope of this study to modify the vegetation boundary conditions, previous work has suggested a high sensitivity to vegetation, showing for example, dramatically increased global annual mean temperatures when interactive vegetation is used, compared to fixed vegetation (Loptson et al., 2014).

When compared to the PI, over Africa the new vegetation resulted in: (a) a loss of the desert regions over the present‐day Sahara, primarily because this is ocean in the early Eocene; (b) a latitudinal expansion (relative to the PI) of tropical rainforest across central Africa; and (c) an addition of a large area of tropical rainforest over southern Africa, which is savanna or grassland in the PI (Figure 1c). However, some features remained similar in the early Eocene relative to the PI, such as the region of tropical rainforest across central Africa being bordered by savannah to the north and south, and the Namib Desert (Figure 1c). The impact on precipitation of these three boundary condition changes is discussed below. Soil parameters, including soil dust fields, were given a globally constant value, and (given the lack of paleodata) no lakes were prescribed unless dynamically predicted (Lunt et al., 2017). Concerning greenhouse gas concentrations, the CO_2_ experiments were divided into a set of standard experiments (which all modeling groups should ideally have conducted) and a set of sensitivity experiments (which were optional). All of these were expressed as multiples of the PI simulation, typically with a CO_2_ of 280 ppmv, and were as follows: 3x and 6x the PI for the standard experiments, and 1x, 1.5x, 2x, 4x, and 9x the PI for the sensitivity experiments (Lunt et al., 2017). See Table 1 for which modeling groups conducted which experiments. All other greenhouse gases were kept as PI, the justification for which is given in Lunt et al. (2017). Concerning aerosols, given the rapid development of representation of aerosols in models the experimental design was flexible here and allowed modeling groups to either leave these as PI, treat aerosols interactively (if possible), prescribe aerosols from Herold et al. (2014), or a combination of the above (Lunt et al., 2017). The solar constant and astronomical parameters were kept identical to the PI, the justification for which is again given in Lunt et al. (2017).

**Table 1:** Models Taking Part in DeepMIP, Including Relevant Details and References

Modeling group responsible	Model	Atmospheric resolution (lon x lat)	CO_2_ experiments undertaken	Run length (years)	References
University of Michigan, US	CESM1.2_CAM5	2.5° × 1.89°	1x, 3x, 6x, 9x	2,000	Hurrell et al. (2013)
Alfred Wegener Institute, Germany/Polish Academy of Sciences, Poland	COSMOS‐landveg_r2413	3.75° × 3.71°	1x, 3x, 4x	9,500	Jungclaus et al. (2006)
Stockholm University, Sweden	GFDL_CM2.1	3.75° × 3.05°	1x, 2x, 3x, 4x, 6x	6,000	Delworth et al. (2006)
University of Bristol, UK	HadCM3B_M2.1aN	3.75° × 2.5°	1x, 2x, 3x	7,800	Valdes et al. (2017)
University of Bristol, UK	HadCM3BL_M2.1aN	3.75° × 2.5°	1x, 2x, 3x	7,800	Valdes et al. (2017)
National Academy of Sciences, Russia	INM‐CM4‐8	2° × 1.5°	6x	1,050	Volodin et al. (2018)
Laboratoire des Sciences du Climat et de l'Environnement, France	IPSLCM5A2	3.75° × 1.89°	1.5x, 3x	4,000	Sepulchre et al. (2020)
University of Tokyo, Japan	MIROC4m	2.8125° × 2.79°	1x, 2x, 3x	5,000	Chan et al. (2011)
University of Bergen, Norway	NorESM1_F	2.5° × 1.89°	2x, 4x	2,100	Guo et al. (2019)

Lastly, the experimental design provided some advice on practical matters such as simulation length and output format. The simulations varied in length (see Table 1) but were all at least 1,000 yr in length, with the climatologies, comprising the results discussed here, being calculated over the final 100 yr. At that point, all simulations should have had a global mean top‐of‐the‐atmosphere net radiation balance of less than 0.3 W m^−2^ (or a similar balance to that of the PI) and an SST trend of less than 0.1°C century^−1^ (Lunt et al., 2017). All of the output, details of which are given in Lunt et al. (2017), were uploaded to a centralized DeepMIP database.

### 2.2 Models

Extensive details on each model, and how the experimental design was implemented in their simulations, are given in Lunt et al. (2021) and references therein and will therefore only briefly be discussed here; those aspects likely to affect precipitation (e.g., convection and land‐surface schemes) will be focused upon here. In total, nine models were included in DeepMIP, although it should be noted that two of these are different configurations of the same model. See Table 1 for a list of the models, along with their atmospheric spatial resolutions and appropriate references (particularly relating to the atmospheric component of the models and elements relating to hydroclimate, where available). In detail, these are as follows.

1. CESM1.2_CAM5: The Community Earth System Model version 1.2 (CESM1.2) is comprised of the Community Atmosphere Model version 5.3 (CAM5), the Community Land Model version 4.0, the Community Ice Code version 4.0, and the Parallel Ocean Program version 2 (Hurrell et al., 2013). CAM5 uses the finite‐volume dynamical core and physical parameterizations of deep convection (G. J. Zhang & McFarlane, 1995), shallow convection and moist turbulence (Park & Bretherton, 2009), and cloud microphysics (Morrison & Gettelman, 2008). This version contains new physical parameterizations in the atmosphere, such as the cloud microphysics, which is critical for the simulation of the large‐scale climate features of the early Eocene (W. Liu et al., 2017).
2. COSMOS‐landveg_r2413: For an atmospheric general circulation model, ECHAM5 (the European Centre Hamburg Model) is used (Roeckner et al., 2003), and this is coupled to the Max‐Planck‐Institute for Meteorology Ocean Model (Marsland et al., 2003); the coupled model is described by Jungclaus et al. (2006). COSMOS‐landveg_r2413 simulates cumulus convection using a mass flux scheme. The orography is represented in spectral domain by surface geopotential (see Stepanek & Lohmann, 2012 for more details regarding model description). The land surface conditions for each biome are based on Hagemann (2002); additionally, parameters with a seasonal cycle (i.e., leaf area index and vegetation ratio) in the latitude belt of ∼20°S–20°N were smoothed and an annual average for each biome was prescribed.
3. GFDL_CM2.1: This uses the Geophysical Fluid Dynamics Laboratory (GFDL) CM2.1 model (Delworth et al., 2006), with modifications as described in Hutchinson et al. (2018), and comprising the Atmosphere Model 2, Land Model 2 and the Sea Ice Simulator 1, coupled to the ocean component from the modular ocean model version 5.1 (MOM5.1). The atmosphere uses a finite‐volume discretization, and a 3° latitude x 3.75° longitude resolution with 24 vertical levels, following the configuration of CM2Mc (Galbraith et al., 2011). Convection is parameterized by the relaxed Arakawa‐Schubert scheme of Moorthi and Suarez (1992), with a lower‐bound on entrainment as specified in Tokioka et al. (1988). Cloud microphysics are parameterized using the scheme of Rotstayn (1997), while cloud macrophysics use the parameterization of Tiedtke (1993). Full details of the convection and cloud parameterizations are given in Delworth et al. (2006). Of possible relevance to the simulation of precipitation, the topography is smoothed using a three‐point mean filter to allow a smoother interaction with the wind field (Lunt et al., 2021).
4. HadCM3B_M2.1aN: This Hadley Centre Climate Model (HadCM3) version is documented extensively in Valdes et al. (2017). In particular, the model uses a single “bulk” cloud model to parameterize dry as well as shallow and deep moist convection (Grant, 1998). The cloud scheme uses a statistical parametrization via a probability density function over the grid‐box total water content (Bushell, 1998). Six short‐wave and eight long‐wave radiation bands are represented by the scheme of Edwards and Slingo (1996). Static fields for the nine surface types of the MOSES2.1 land surface scheme (Cox et al., 1999) are derived from the 10 megabiomes of the DeepMIP vegetation boundary conditions (Herold et al., 2014) via a lookup table. The atmosphere uses a Cartesian grid with a horizontal resolution of 3.75 × 2.5° (longitude x latitude) and 19 hybrid vertical levels.
5. HadCM3BL_M2.1aN: The only difference between this version of HadCM3 and the one described above is the horizontal resolution of the ocean component (Cox, 1984), at 1.25° × 1.25° for HadCM3B_M2.1aN and 3.75° × 2.5° for HadCM3BL_M2.1aN, and associated diffusion parameters (Valdes et al., 2017). Both versions use 20 unequally spaced vertical levels in the ocean ranging between 10 and 616 m.
6. INM‐CM4‐8: This version of the Institute of Numerical Mathematics (INM) model is described in Volodin et al. (2018), but the parameterizations of physical processes are the same as in the previous version, INM‐CM5, and described more detail in Volodin et al. (2017). Parameterization of condensation and cloud formation follows Tiedtke (1993), and cloud water is a prognostic variable. Parameterization of cloud fraction follows Smagorinsky (1963); cloud fraction is a diagnostic variable, independent of the calculation of condensation, and depended on the relative humidity. The surface, soil, and vegetation scheme follow Volodin and Lykossov (1998), with the evolution of the equations for temperature, soil water, and soil ice being solved at 23 levels from the surface to 10 m depth (Volodin et al., 2018). The fractional area of 13 types of potential vegetation is specified, and actual vegetation as well as LAI is calculated according to the soil water content in the root zone and soil temperature (Volodin et al., 2018).
7. IPSLCM5A2: The IPSL‐CM5A2 Earth system model from the Institut Pierre Simon Laplace (IPSL) is documented by Sepulchre et al. (2020), and is based on the previous generation IPSL Earth system model (IPSLCM5A, Dufresne et al., 2013) but with new revisions such as a re‐tuning of global temperature. It comprises the LMDZ5 (Laboratoire de Météorologie Dynamique Zoom) atmosphere model, the Organizing Carbon and Hydrology In Dynamic Ecosystems (ORCHIDEE) land surface and vegetation model and the Nucleus for European Modeling of the Ocean (NEMOv3.6) ocean model, which includes the LIM2 sea ice model and the Pelagic Interactions Scheme for Carbon and Ecosystem Studies (PISCESv2) biogeochemical model (Lunt et al., 2021). LMDZ5 runs at a horizontal resolution of 1.9° × 2.5° (latitude × longitude) with 39 hybrid sigma‐pressure levels. The LMDZ5 radiation scheme is inherited from the European Center for Medium‐Range Weather Forecasts (Fouquart & Bonnel, 1980; Morcrette et al., 1986), and the dynamical effects of the subgrid‐scale orography are parameterized according to Lott (1999). Turbulent transport in the planetary boundary layer is treated as a vertical eddy diffusion (Laval et al., 1981), with counter‐gradient correction and dry convective adjustment, and the surface boundary layer is treated according to Louis (1979). Cloud cover and cloud water content are computed using a statistical scheme (Bony & Emanuel, 2001). For deep convection, the LMDZ5A version uses the “episodic mixing and buoyancy sorting” scheme originally developed by Emanuel (1991).
8. MIROC4m: This version of the Model for Interdisciplinary Research on Climate (MIROC) is documented by K‐1 model developers (2004) and summarized in Chan et al. (2011). In the atmosphere model, cumulus parameterization is based on Arakawa and Schubert (1974), with some simplifications and the cloud base mass flux is treated as a prognostic variable. Cumulus convection is suppressed when the cloud‐mean ambient relative humidity is less than the critical value of 0.8. The land surface model (Minimal Advanced Treatments of Surface Interaction and Runoff, MATSIRO) is documented by Takata et al. (2003), where prognostic variables include canopy temperature, canopy water content, snow amount, soil moisture content, and frozen soil moisture content. Fixed vegetation types are specified over ice‐sheet‐free. The ocean component is version 3.4 of the CCSR (Center for Climate System Research) Ocean Component Model (COCO), documented in Hasumi (2000).
9. NorESM1_F: This version of the Norwegian Earth System Model (NorESM) is described in detail in Guo et al. (2019) and Li et al. (2020), and differs from the previous version (NorESM1‐M) in that while it has the same atmosphere‐land grid, the ocean and sea ice components use a tripolar grid (rather than the bipolar grid in NorESM1‐M), resulting in a more realistic Atlantic Meridional Overturning Circulation (Lunt et al., 2021). NorESM1_F couples the Miami Isopycnic Coordinate Ocean Model (MICOM) and the spectral Community Atmosphere Model (CAM4) (Eaton, 2010; Neale et al., 2008, 2013). CAM4 includes the G. J. Zhang and McFarlane (1995) deep convection scheme, the Hack (1994) shallow convection scheme, the nonlocal boundary layer scheme of Holtslag and Boville (1993) and the representation of cloud microphysics and macrophysics by Rasch and Kristjánsson (1998) and M. Zhang et al. (2003). Instead of using the undiluted convective available potential energy (CAPE) in the original deep convection scheme, the diluted CAPE through an explicit representation of entrainment has been used to close the cumulus parameterization (Neale et al., 2008). The convective momentum transport has also been included in the parameterization of deep convection (Richter & Rasch, 2008). Additionally, NorESM1_F adopts energy updates and energy conservation. Compared to NorESM1‐M, NorESM1_F has several important improvements on how precipitation is simulated, such as improvements in seasonality, a reduced wet bias and mitigation of the common double intertropical convergence zone (ITCZ) problem (Li et al., 2020).

### 2.3 Observational and Proxy Data

Here, the observational and proxy data are described; first there is a description of the modern, satellite‐derived data used to assess and evaluate the PI simulations, and second there is a description of the early Eocene proxy data used to evaluate the Eocene simulations.

#### 2.3.1 Satellite‐Derived Rainfall Estimates From the Modern Period

Even in the 21st century, there is a severe lack of in‐situ rain gauge data over Africa; South Africa is probably the best populated in terms of rainfall measurements, but in other countries such as Angola or Namibia rain gauge data are sparse or non‐existent (e.g., Williams et al., 2007, 2008, 2010). The CenTrends precipitation data set (Funk et al., 2015) contains measurements going back to 1900, but only for a small number of countries in East Africa. Likewise, although the Global Historical Climate Network database (Durre et al., 2010, 2008; Menne et al., 2012) does contain temperature measurements going back to 1861, precipitation measurements do not begin until the 1950s and are again relatively sparse in Africa. Therefore, a possible solution to the problem of data unavailability is to use satellite‐derived rainfall estimates (SREs), which offer near‐uniform coverage at relatively high spatial resolution from the 1980s onwards.

Several data sets of SREs currently exist, but here the Tropical Applications of Meteorology using SATellite data and ground‐based observations (TAMSAT) is used. TAMSAT (version 3.1) provides daily, 10‐daily, monthly and seasonal precipitation estimates over Africa at 4 km resolution, and extends from 1983 to the present‐day. The data are publicly available; please see Open Research section, and Maidment et al. (2014, 2017) and Tarnavsky et al. (2014) for details. Here, TAMSAT is used as a comparative tool for evaluating the PI simulations of the DeepMIP models. A caveat here is that the models are showing precipitation simulated under PI boundary conditions, whereas TAMSAT is showing precipitation from the late 20th and early 21st century (referred to here as modern) and will therefore contain an anthropogenic signal; this, however, is unavoidable given the lack of PI precipitation observations. It is expected that the biases between comparing the models to PI precipitation vs. comparing them to modern precipitation will be less than the biases between the models themselves (i.e., the inter‐model spread), and indeed much less than the uncertainty associated with the Eocene reconstructions.

#### 2.3.2 Paleobotanical Eocene Precipitation Estimates

The distribution and physiognomy of land plants are sensitive to precipitation (Wright et al., 2017). Therefore, the taxonomic affinity and the morphology of leaf fossils can be used to generate paleo‐precipitation estimates (e.g., Utescher et al., 2014; Wilf et al., 1998). For this study, previously established Paleocene‐Eocene (∼41–56 Ma) paleobotanical records from Africa were compiled (see Supporting Information for age ranges for individual sites, Table S1 in Supporting Information S1). The distribution of the nearest living relatives (NLR) of these taxa was then analyzed using the bioclimatic analysis approach to find the highest probability precipitation range in which all taxa could co‐occur (e.g., West et al., 2020; Willard et al., 2019).

Geodetic coordinates of occurrences were obtained for the NLR of each plant group from the Global Biodiversity Information Facility (see Table S2 in Supporting Information S1). These occurrence data sets were then filtered for uncertain, exotic, and superfluous occurrences, as well as subjected to a random resampling to avoid regional overrepresentation of densely sampled areas. A climatic envelope for each plant group (see Table S2 in Supporting Information S1) was then generated by extracting precipitation data (MAP, wettest month (WMP), driest month (DMP), warmest and coldest quarter precipitation (WQP and CQP, respectively) and the precipitation seasonality coefficient [PS]) using the DISMO package in R (Hijmans et al., 2005). A probability density function was then generated for each co‐occurring plant group by testing the likelihood of the plant group occurring at 100,000 unique extant combinations of MAP, WMP, DMP, PS, WQP, and CQP. As shown in Equation 1, the product of probabilities (*f*) was calculated for each plant group (*t*) at each climatic combination (*x*), using the means (*μ*) and standard deviations (*σ*) of their modern‐day bioclimatic envelope, for each climatic variable (*c*).

(1) $$f\left(t_{n}\right)=\overset{6}{\underset{i=1}{\prod }}\frac{1}{\sqrt{2\sigma_{c}^{2}\times \pi }}e^{x_{c}-\mu_{c}/2\sigma_{c}^{2}}$$

A combined likelihood for all plant groups in the assemblage combined can then be calculated with the product of all likelihoods (*n*), shown in Equation 2.

(2) $$f\left(z\right)=\overset{n}{\underset{i=1}{\prod }}t_{n}$$

The combination of MAP, WMP, DMP, PS, WQP, and CQP with the highest likelihood is the value reported here as most representative for the assemblage, and the highest and lowest values of the metrics with *f*(*z*) ≥ 5% of the maximum *f*(*z*) is represented as the uncertainty (using the 95% confidence interval).

Eleven plant assemblages from South Africa, Tanzania, South Sudan, Cameroon, Côte d’Ivoire, Ghana, and Nigeria were analyzed with the bioclimatic analysis NLR method (Adeonipekun et al., 2012; Atta‐Peters & Salami, 2004; Cantrill et al., 2013; Chiaghanam et al., 2017; de Villiers, 1997; Eisawi & Schrank, 2008; Goha et al., 2016; Okeke & Umeji, 2016; Salami, 1984; Salard‐Cheboldaeff, 1979; Uzodimma, 2013); see Table S1 in Supporting Information S1 for age ranges of individual sites.

The NLR generated precipitation values were supplemented with an additional value based on leaf area analysis derived data by Jacobs and Herendeen (2004) and Kaiser et al. (2006), also from Tanzania (from the Lutetian). In locations where the final results are in the same geographical location, the reconstructions were averaged. The final results of this analysis are shown in Table 2, with Eocene MAP expressed as ranges and modern MAP taken from TAMSAT. It should be noted that, for the results other than the model‐data comparison, precipitation during June–August (JJA) is focused upon, rather than using MAP. Previous work has suggested that for much of the continent, over 80% of the annual total of precipitation is accounted for by a given region's wet season and, over West Africa (the wet season of which is JJA), this increases to 95% or higher (Liebmann et al., 2012). Given that the primary driver of this wet season is the seasonal progression of the ITCZ, it is likely that this relationship will hold during the early Eocene. It was therefore deemed appropriate to focus on this season for the majority of the analysis (i.e., Sections 3.1, 3.2 and 3.3), only using MAP for the model‐data comparison (Section 3.4), for which seasonal proxy data are not available.

**Table 2:** Locations and Mean Annual Precipitation (MAP) From Early Eocene Paleobotanical Records From Africa, and Modern Values

Site name	Latitude (°N)	Longitude (°E)	MAP (mm yr^−1^)	
			Early Eocene	Modern
Koningsnaas, South Africa	−30.2	17.3	1,318–1,738	101
Shagamu, Nigeria	6.7	3.7	1,148–2,089	1,762
Melut Basin, South Sudan	10	33	1,175–1,905	757
Kwakwa, Cameroon	4.5	9.1	1,175–1,905	2,524
Mwadui, Tanzania	−3.9	33.5	813–1,738	754
Tano, Ghana	4.7	−3	1,514–2,344	‐
Nanka, Nigeria	6.12	7	1,380–2,291	1,683
Abidjan margin, Côte d’Ivoire	5	−4.1	1,660–1,950	‐
Okigwe, Nigeria	5.82	7.34	1,175–1,862	2,311
Bende ‐ Umuahia, Nigeria	5.47	7.45	1,514–2,291	2,311
Araromi, Nigeria	7.7	3.5	1,072–1,738	1,179
Mahenge, Tanzania	−4.79	34.26	720–800	707
Mahenge, Tanzania	−4.79	34.26	630–690	707
Mahenge, Tanzania	−4.79	34.26	737–815	707
Mahenge, Tanzania	−4.79	34.26	644–708	707
Mahenge, Tanzania	−4.79	34.26	710–790	707
Mahenge, Tanzania	−4.79	34.26	610–680	707
Mahenge, Tanzania	−4.79	34.26	610–680	707
Mahenge, Tanzania	−4.79	34.26	740–820	707

*Note.* Early Eocene ranges of MAP are expressed as the 95% confidence interval for all locations except Mahenge, where ranges are expressed as +/−1 standard deviation. Modern values of MAP taken from TAMSAT; missing values indicate ocean regions, as TAMSAT MAP is land only.

## 3 Results

Here, the results of different comparisons are described: (a) a model validation exercise, where the models' PI simulations are compared to modern observations (Section 3.1); (b) a simulation comparison, where precipitation from the models' early Eocene simulations, at varying levels of CO_2_, is compared (Section 3.2); (c) a simulation comparison, where the physical mechanisms behind the precipitation response are investigated (Section 3.3); and (d) a model‐data comparison, where precipitation from the models' early Eocene simulations is compared to available proxy data (Section 3.4).

### 3.1 DeepMIP Models' Preindustrial Simulations vs. Modern Observations

**Figure 2:**
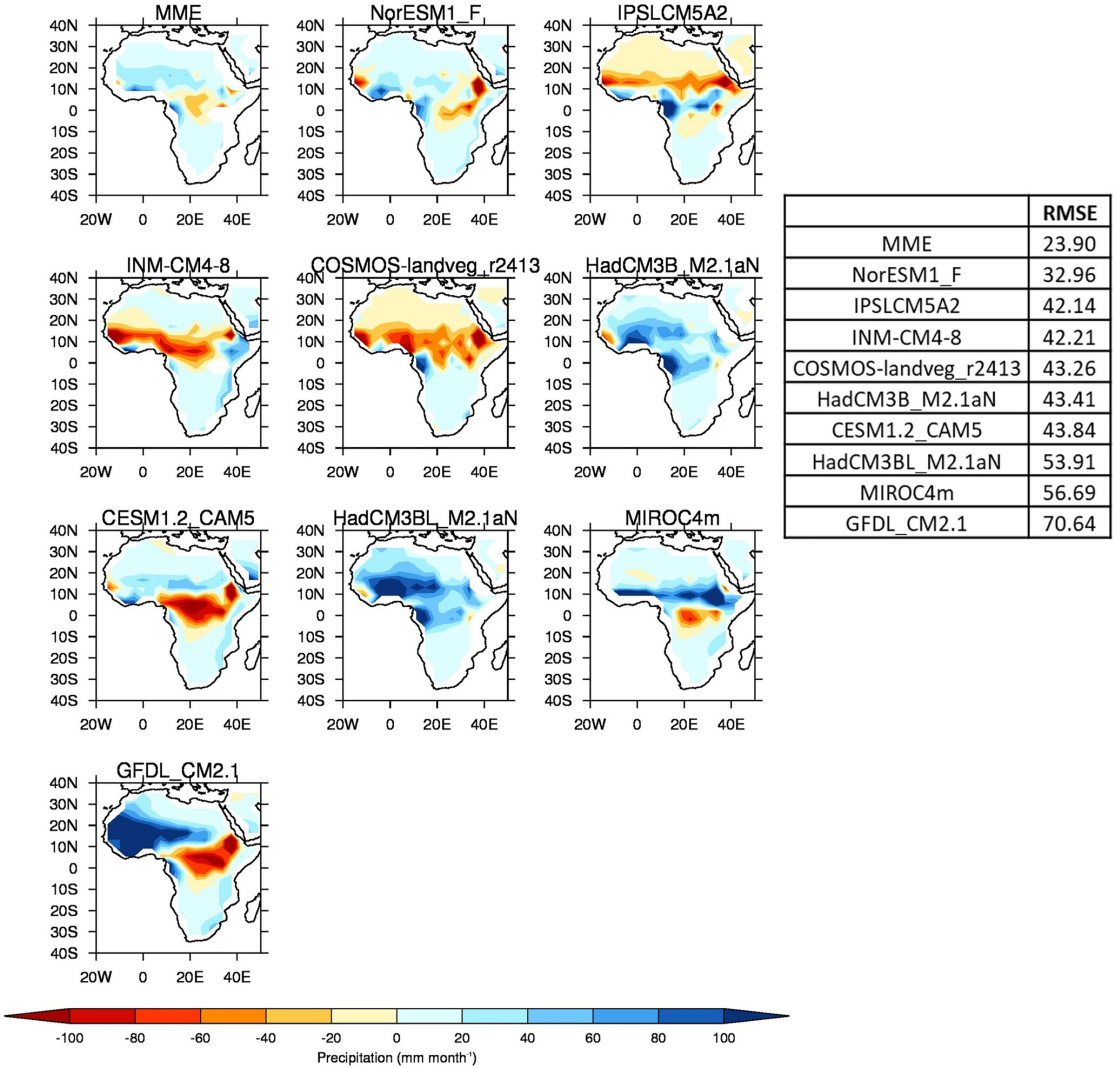
June‐August (JJA) precipitation climatology differences (PI simulations‐TAMSAT), re‐gridded to lowest common spatial resolution (that of COSMOS‐landveg_r2413) and ordered according to root mean squared error (RMSE, in mm month^−1^, see insert). RMSE calculated over 20°W–50°E, 40°N–40°S, land points only.

Here, the focus is on mean precipitation differences between the various DeepMIP PI simulations (including the multi‐model ensemble mean, MME) and precipitation observations from TAMSAT (see Section 2.3.1). Here, the MME is calculated for a given variable as the simple mean across all available models. Precipitation anomalies (PI simulations‐TAMSAT) during JJA are shown in Figure 2, where the models have been ordered according to the root mean square error (RMSE), relative to TAMSAT. Two observations are noteworthy. First, the MME is showing by far the closest agreement to TAMSAT, with a much lower RMSE (by ∼10 mm month^−1^ less than even the next lowest individual model), highlighting the importance of using the MME to counterbalance individual models' biases (whether they be under or overestimating). The MME will therefore subsequently be used when discussing the various Eocene simulations. Second, there appears to be a divide between: (a) models such as IPSLCM5A2, INM‐CM4‐8, and COSMOS‐landveg_r2413 that are underestimating African precipitation (i.e., are showing drier conditions across West Africa at ∼10°N), which have relatively low RMS error compared with TAMSAT; and (b) models such as HadCM3BL_M2.1aN, MIROC4m and GFDL_CM2.1 that are overestimating African precipitation, which have relatively high RMS error compared with TAMSAT. For example, the model with the least agreement (GFDL_CM2.1, RMSE = 70.6 mm month^−1^) is overestimating precipitation over West Africa by more than 100 mm month^−1^.

**Figure 3:**
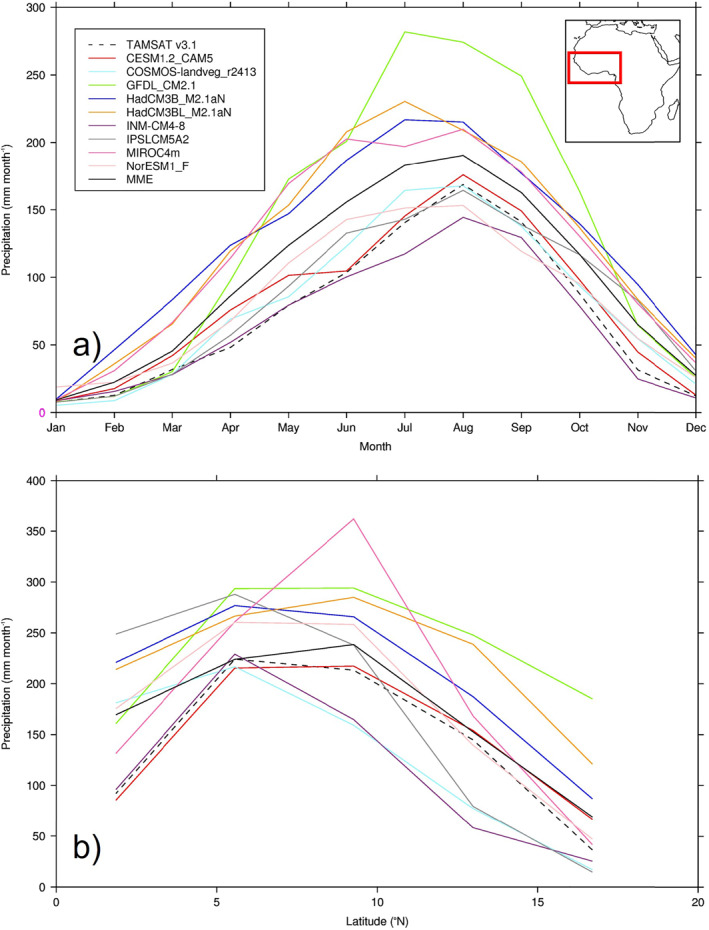
Precipitation climatology from TAMSAT and PI simulations, averaged over West Africa (20°W–15°E, 0°–20°N‐land points only): (a) Mean seasonal cycle, at each model's individual spatial resolution; (b) Zonal mean of June–August (JJA) precipitation, re‐gridded to lowest common spatial resolution.

Concerning the seasonal and latitudinal distribution of African precipitation, Figure 3 shows the annual cycle of West African (defined here as land points only encompassing 20°W–15°E, 0°–20°N) precipitation and the zonal mean of JJA West African precipitation (Figures 3a and 3b, respectively). Outside of JJA, the majority of models are overestimating precipitation throughout the year (Figure 3a), with the model closest to TAMSAT (in terms of the seasonal cycle i.e., precipitation timings) being CESM1.2_CAM5, although even this model overestimates precipitation during the first half of the year. When averaged over this region, only one model (INM‐CM4‐8) underestimates precipitation throughout the year, but is nevertheless closer to TAMSAT than those which overestimate, in agreement with that discussed above and shown in Figure 2. One model (GFDL_CM2.1) greatly overestimates precipitation especially during JJA, and others (such as INM‐CM4‐8) underestimate precipitation during JJA and therefore do not correctly reproduce the strong seasonality (i.e., the precipitation curve is flatter); for example, the difference between the wettest and driest month in this model is 136 mm month^−1^, whereas it is 161 mm month^−1^ in TAMSAT and 181 mm month^−1^ in the MME (Figure 3a). The MME also overestimates precipitation throughout the year but is nevertheless closer to TAMSAT in terms of seasonality than many of the wetter models (Figure 3a). Latitudinally, most models are showing a much wider (in terms of latitudinal extent) rain belt relative to TAMSAT, with GFDL_CM2.1 and the HadCM3 family in particular not reproducing the observed rapid drop‐off in precipitation either near the Equator or north of 15°N (Figure 3b). In part due to some drier models approaching the Equator (such as CESM1.2_CAM5 and INM‐CM4‐8), the MME is showing a similar latitudinal extent of precipitation compared to TAMSAT, and while it is still too wet at low latitudes it does correctly drop off north of 15°N (Figure 3b).

### 3.2 DeepMIP Models' Early Eocene Simulations Relative to Preindustrial Simulations and Each Other

Here, the focus is on mean precipitation differences between various DeepMIP early Eocene CO_2_ sensitivity experiments, in which all boundary conditions other than CO_2_ were kept identical. The focus is not only on the precipitation response to varying CO_2_ concentrations relative to the PI simulations, but also from each CO_2_ experiment individually (relative to each other). Precipitation anomalies of all the CO_2_ experiments vs. PI are first briefly presented (Section 3.2.1), and then the experiment results are divided into a CO_2_ component (Section 3.2.2) and a non‐CO_2_ component (i.e., the impact of the other boundary condition changes, Section 3.2.3).

Before these results are presented, however, a brief introduction to the early Eocene precipitation over Africa is needed. Mean JJA precipitation over PI and early Eocene Africa (using the 1x CO_2_ simulation) is shown in the Supporting Information, where it is clear that, during the PI, all models are showing a band of precipitation between approximately the Equator and 10°N that extends from the central equatorial Atlantic all the way across Africa (Figure S2a in Supporting Information S1). How far east this rain belt extends is dependent on model, but the majority (and the MME) show it extending up to 40°E. During the early Eocene, although this rain belt is still present over West Africa, most models agree that it does not extend across the continent, instead ending at ∼20°E and being replaced by relatively drier conditions (Figure S2b in Supporting Information S1). Wetter conditions are shown further east, over the early Eocene Indian Ocean, but concerning Africa these results would suggest that although the rain belt is latitudinally similar to the PI, it does not have the longitudinal extent.

#### 3.2.1 All CO_2_ Experiments vs. Preindustrial

**Figure 4:**
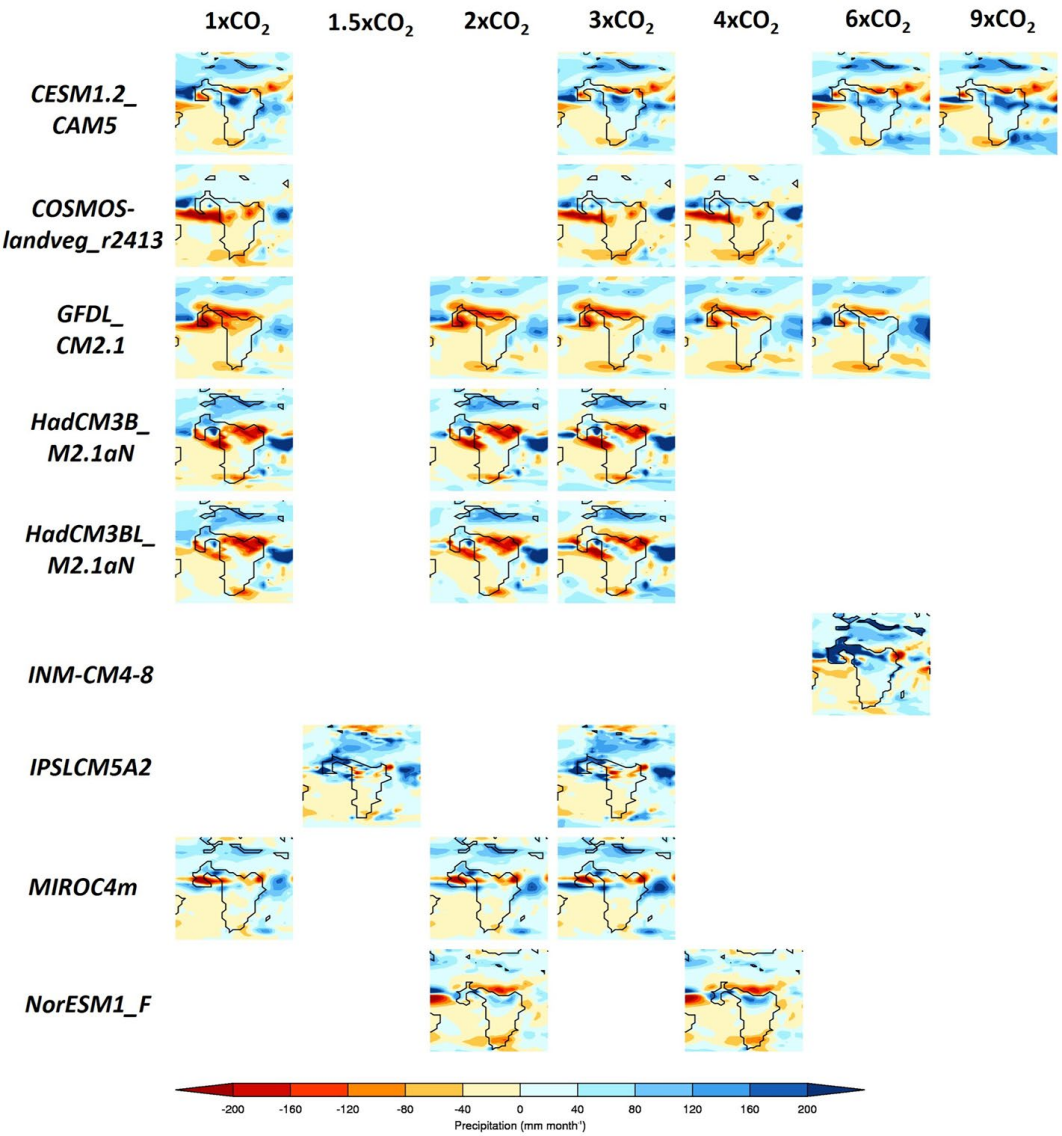
June–August (JJA) precipitation climatology differences (early Eocene‐PI), for each CO_2_ simulation from each model.

The precipitation anomalies (early Eocene‐PI), for each CO_2_ experiment and for each model during JJA are shown in Figure 4. This is only briefly presented, because the combination of a paleogeographic forcing and a CO_2_ forcing makes interpretation difficult; this is why the results are broken down into a CO_2_ component and non‐CO_2_ component below. It should be noted that when the MME is discussed below (see Sections 3.2.2 and 3.2.3), only models that participated in the particular experiment are included.

There is no clear linear trend in either wetting or drying across early Eocene Africa as the CO_2_ concentrations increase (Figure 4). Although many models show drying (relative to the PI) of up to ∼180 mm month^−1^ across northern and western Africa in the 1x, 2x, and 3x experiments, this gradually disappears as higher CO_2_ concentrations are applied, with some models showing precipitation increases of over 200 mm month^−1^ (Figure 4). Some models disagree regardless of experiment, such as GFDL_CM2.1 which shows drying over northern Africa in all CO_2_ experiments contrasting with IPSLCM5A2 which shows wetting over northern Africa in all CO_2_ experiments. Further south, none of the models in any of the experiments are showing a large precipitation response. In very general terms, however, at the lower levels of CO_2_ concentrations (i.e., up to 4x) the majority of models are showing the same region of drying over northern and western Africa.

#### 3.2.2 Lower and Higher CO_2_ Experiments: Impact of CO_2_

To investigate the impact of increasing CO_2_ on precipitation, when all other boundary conditions are constant, the experiments have been divided into two samples, each containing a different number of models going into the MME: (a) “lower‐level CO_2_”, namely the 1x, 2x, and 3x experiments, comprising four models (GFDL_CM2.1, HadCM3B_M2.1aN, HadCM3BL_M2.1aN, and MIROC4m); and (b) “higher‐level CO_2_”, namely the 3x and 6x experiments, comprising two models (CESM1.2_CAM5 and GFDL_CM2.1); see Table 1. Note that the MMEs for the two 3x experiments are slightly different because they contain a different number of models. Here, both absolute precipitation values and anomalies are shown, where the anomalies are of a certain CO_2_ experiment vs. another CO_2_ experiment, rather than early Eocene vs. PI.

**Figure 5:**
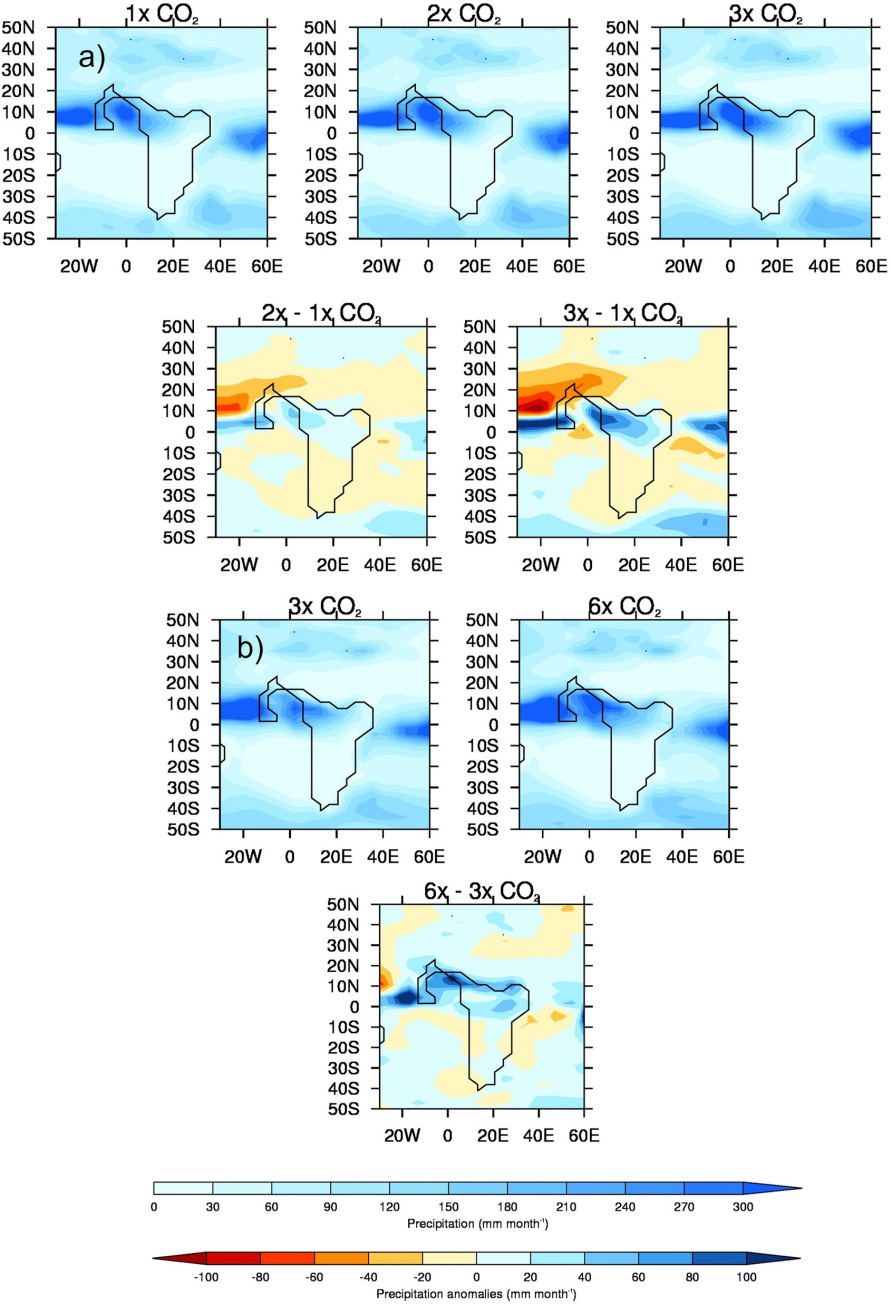
JJA precipitation multi‐model ensemble mean (MME) climatology absolutes and anomalies for the 1x, 2x, 3x, and 6x CO_2_ experiments, using both samples: (a) Lower‐level sample of CO_2_ experiments (comprising the four models that conducted these: GFDL_CM2.1, HadCM3B_M2.1aN, HadCM3BL_M2.1aN, and MIROC4m), absolutes (top row) and anomalies (second row); (b) Higher‐level sample of CO_2_ experiments (comprising the two models that conducted these: CESM1.2_CAM5 and GFDL_CM2.1), absolutes (top row) and anomalies (second row).

The MME absolute precipitation and anomalies for the lower‐level sample of CO_2_ experiments, are shown in Figure 5a. When the absolute values are considered (Figure 5a, top row), all experiments show regions of precipitation maxima over the equatorial Atlantic (north of the Equator) and West Africa. Over the same West African region as described above (20°W–15°E, 0°–20°N, land points only), mean JJA precipitation is 192, 201, and 207 mm month^−1^ for the 1x, 2x, and 3x experiments, respectively, implying a small increase as CO_2_ increases. This becomes more evident when the anomalies are considered (Figure 5a, second row). If the 1x and 2x experiments are compared, the largest change is over the equatorial Atlantic, with a small increase in precipitation of up to 50 mm month^−1^ over the Equator and a decrease of over 50 mm month^−1^ further north, suggesting a southward displacement of the Atlantic ITCZ. Precipitation is also increased over western Africa. The same pattern is evident when the 1x and 3x experiments are compared, but more pronounced, with both the increases and decreases approaching 100 mm month^−1^ in their respective areas.

The MME absolute precipitation and anomalies for the higher‐level sample of CO_2_ experiments are shown in Figure 5b. When the absolute values are considered (Figure 5b, top row), the region of precipitation maxima in the equatorial Atlantic is larger in the 6x experiment. Over the same West African region, mean JJA precipitation is 186 and 232 mm month^−1^ for the 3x and 6x experiments, respectively, implying a large mean increase as CO_2_ increases, and this is further confirmed when the anomalies are considered (Figure 5b, second row). Precipitation increases of over 100 mm month^−1^ are shown over the equatorial Atlantic (north of the Equator) and West Africa in the 6x relative to the 3x experiment, but the large region of drying seen at the lower levels of CO_2_ is less evident (Figure 5b, second row). This suggests that, whilst West African precipitation is still (and more so here) enhanced as CO_2_ rises, it is perhaps less related to Atlantic ITCZ displacement and more related to an increase in south‐westerly flow (discussed further in Section 3.3).

#### 3.2.3 1x CO_2_ Experiment vs. Preindustrial: Impact of Non‐CO_2_ Boundary Conditions

The 1x CO_2_ experiment vs. PI is of particular interest, because this shows the impact of the other boundary conditions rather than that from CO_2_ concentrations. When CO_2_ concentrations are kept as PI (as in the 1x experiment), the boundary conditions (see Section 2.1) likely to have the largest impact on regional precipitation are the LSM, topography, and vegetation (see Figure 1). Although land ice changes, the largest of which during the early Eocene were over the Antarctic Ice Sheet (AIS), do cause a precipitation response (e.g., Kennedy‐Asser et al., 2019), this is thought to be a mainly local signal and further afield, such as over northern and western Africa during JJA, there is little or no precipitation change when the AIS is either imposed or removed (Kennedy‐Asser, pers. comm.).

**Figure 6:**
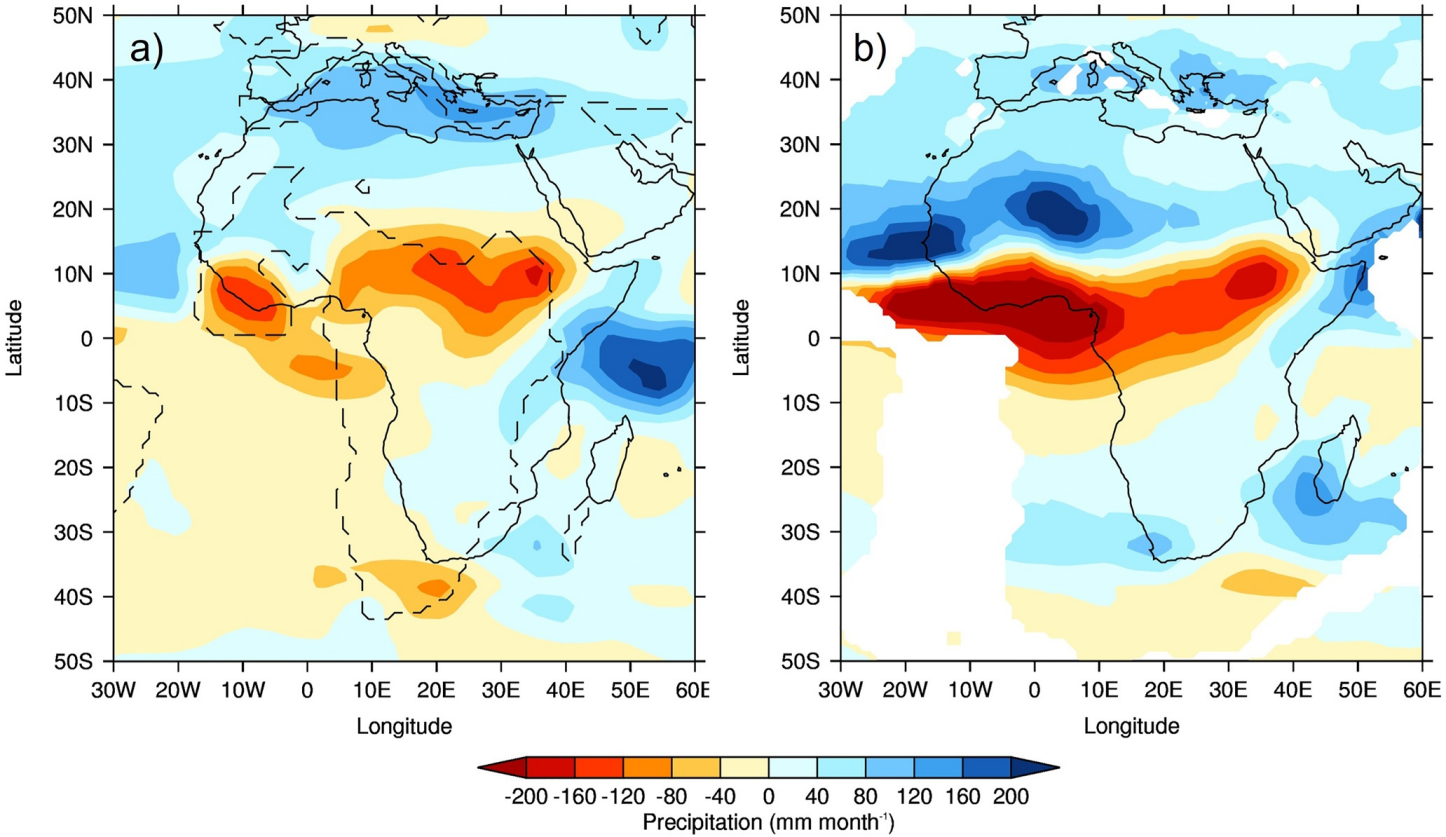
June–August (JJA) precipitation multi‐model ensemble mean (MME) climatology differences (early Eocene‐PI) for the 1x CO_2_ experiment (comprising the four models that conducted this experiment, in addition to the others considered here: GFDL_CM2.1, HadCM3B_M2.1aN, HadCM3BL_M2.1aN, and MIROC4m): (a) Original (i.e., unrotated) differences; (b) Rotated differences that is, Charlie Eocene precipitation rotated forward to where it is in the PI. Note that in (a), solid lines show the PI mask and dashed lines show the Eocene mask.

The MME precipitation anomaly for this experiment is shown in Figure 6a; it should be noted that, although six models conducted this experiment (CESM1.2_CAM5, COSMOS‐landveg_r2413, GFDL_CM2.1, HadCM3B_M2.1aN, HadCM3BL_M2.1aN, and MIROC4m), only the latter four are included here in the MME, to be consistent with the analysis of the CO_2_ component (Section 3.2.2). From the available DeepMIP results, it is impossible to disentangle the boundary conditions and ascertain which is dominant in causing the precipitation response; in an ideal world, sensitivity experiments would be conducted to introduce each boundary condition individually, but this is not possible with the results currently available on the DeepMIP database. Nevertheless, based on the results it is possible to theorize which of these boundary conditions might be causing this MME precipitation response. The largest precipitation changes relative to the PI are a small increase in precipitation to the north of early Eocene Africa and in the western Indian Ocean, and a decrease in precipitation over western and northern equatorial Eocene Africa (Figure 6a). It is likely that the northern increases are caused by the change in the LSM (Figure 1a) as this region comprises the preindustrial (and modern) Sahara but is ocean in the early Eocene and therefore would have been a much greater moisture source. Likewise, the increase over the western Indian Ocean is likely because preindustrial Africa extends much further East than during the early Eocene, again giving much less of a moisture source during the PI (Figure 1a). Moreover, an examination of SST from the early Eocene and PI simulations (from each individual model and the MME) shows that these exposed regions of ocean are characterized by warmer SSTs in the early Eocene; for example, in the Indian Ocean absolute values are up to 32°C in the early Eocene MME compared to up to 28°C in the PI MME, thereby providing a greater source of evaporation during the Eocene see (see Figure S3 in Supporting Information S1). Concerning the drying over equatorial early Eocene Africa, this is more difficult to interpret and does not seem likely to be related to the LSM or the changes in vegetation. For the LSM, this region of drying coincides with land during both time periods. For the vegetation, although there is a shift in biome between the PI and early Eocene, the region of drying (at approximately 10°–20°N) coincides with an increase (or slightly northward shift) in tropical rainforest during the early Eocene, rather than mostly being savanna and grassland in the PI (Figure 1c). This might be expected to result in an increase in precipitation during the early Eocene, rather than a drying. However, this response might be explained by the difference in orographic heights over this region (i.e., over central equatorial Africa), where early Eocene Africa is considerably lower (up to 400 m) than in the PI (up to 1,000 m). Finally, over southern Africa, although there is a large increase in orographic heights (of over 1,000 m) during the early Eocene (Figure 1b and Figure S1 in Supporting Information S1), this does not appear to be having a large impact on African precipitation, with minimal precipitation differences in the south (Figure 6a).

However, a caveat of the above analysis is that, because of the plate rotation differences during the early Eocene, Figure 6a is showing precipitation anomalies that may simply be due to differing geographical locations, rather than any change to the climate state. Therefore, Figure 6b shows the same results, but this time with the early Eocene precipitation rotated forwards (based on the rotations supplied in Herold et al., 2014 Supporting Information) to where it is in the PI. However, despite these rotational differences, the overall picture remains the same (i.e., increases in precipitation over northern Africa and a decrease in precipitation over western and equatorial Africa) but much more pronounced (Figure 6b). The increases and decreases in precipitation exceed 200 mm month^−1^ in some places, suggesting a northward displacement of the Atlantic ITCZ; this difference between the early Eocene and the PI is in contrast to when the Eocene CO_2_ experiments are compared with each other, to assess the impact of increasing CO_2_ (discussed previously in Section 3.2.2).

### 3.3 Physical Mechanisms Behind the Precipitation Response

Here, the focus is on the possible dynamic and thermodynamic mechanisms causing the observed precipitation responses, again using the MME absolute values and anomalies from the aforementioned lower‐and higher‐level samples of CO_2_ experiments.

**Figure 7:**
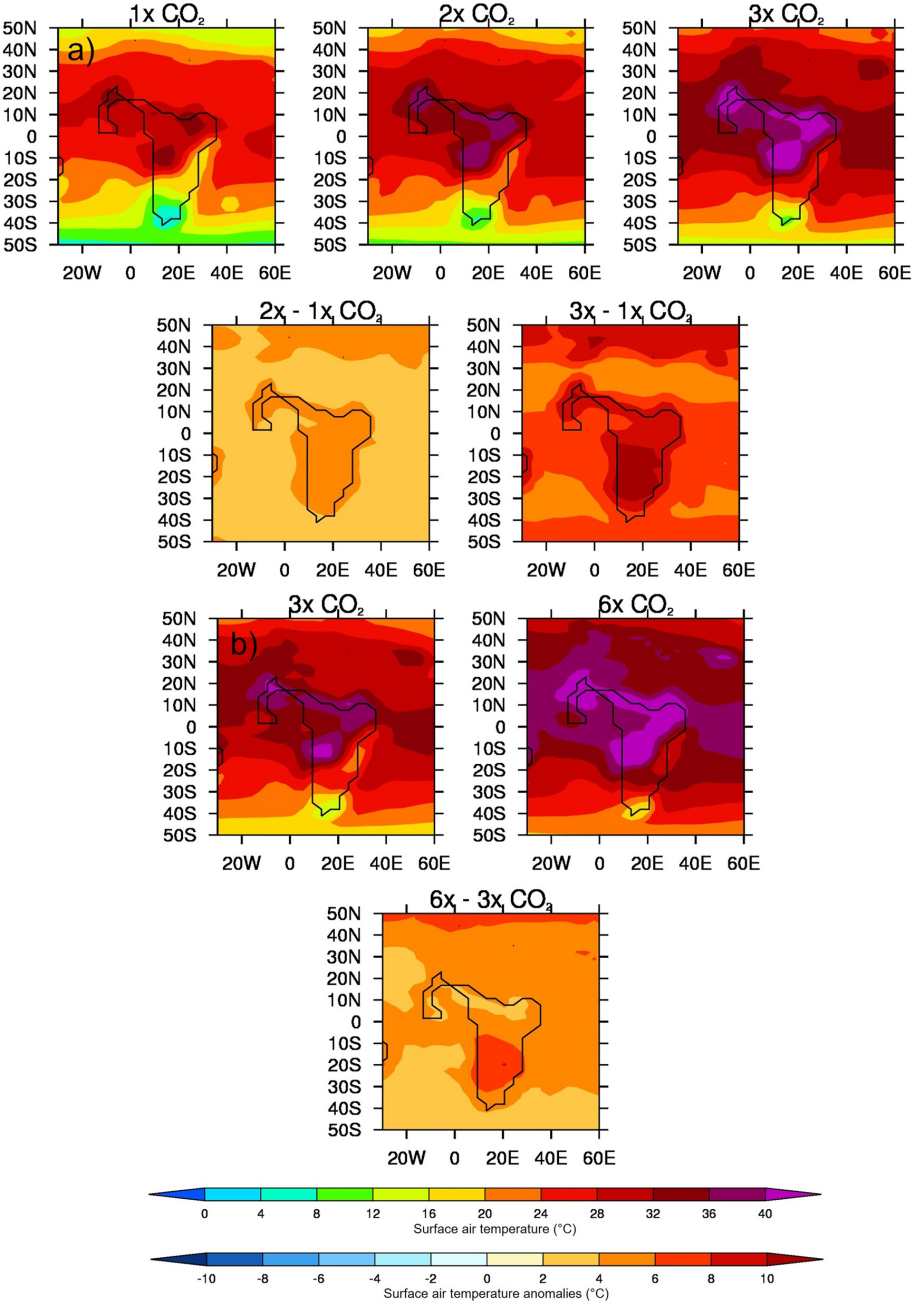
Same as Figure 5 but for June–August (JJA) 1.5 m surface air temperature.

**Figure 8:**
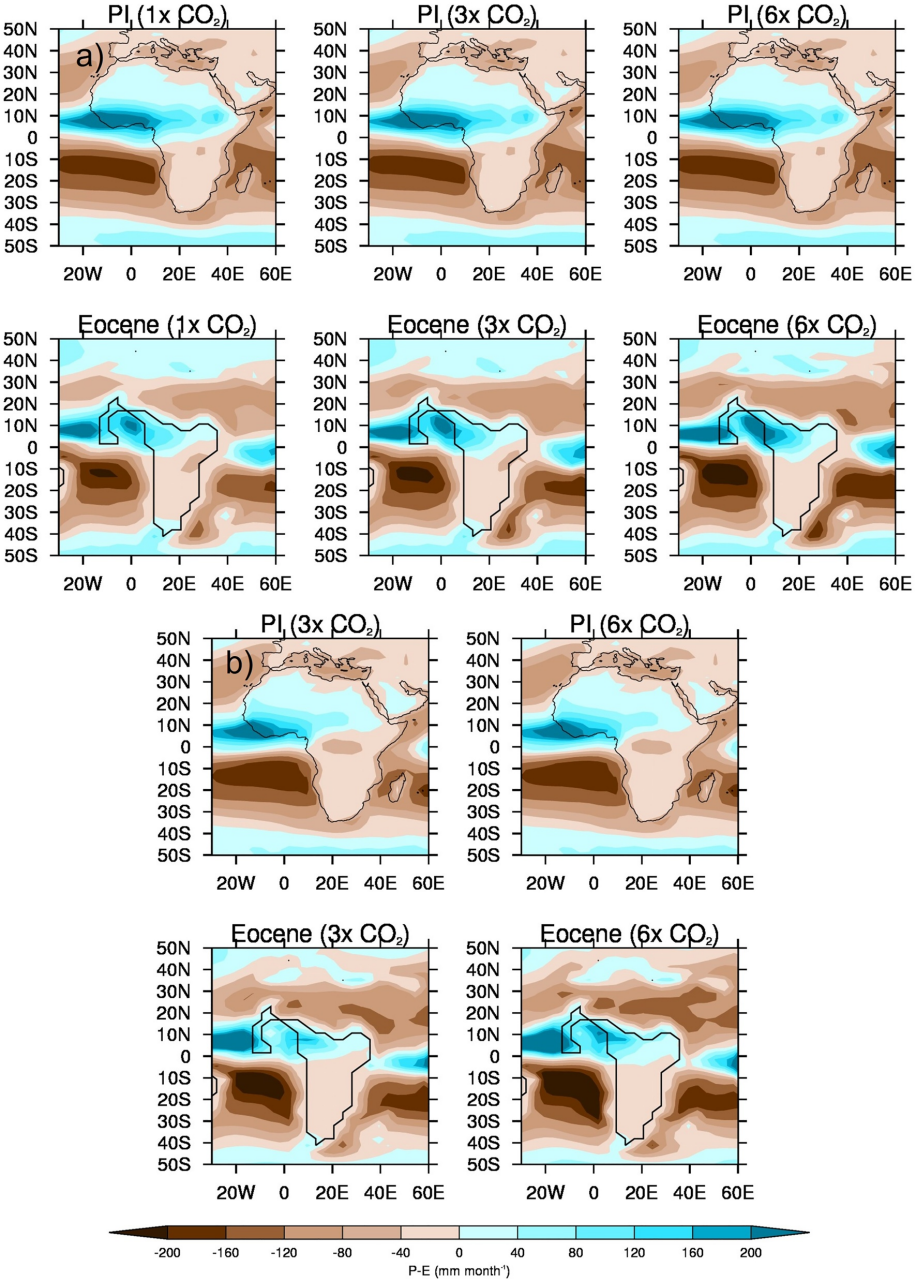
June–August (JJA) precipitation‐evaporation (P‐E) multi‐model ensemble mean (MME) climatology absolutes for the 1x, 2x, 3x, and 6x CO_2_ experiments, using both samples: (a) lower‐level sample of CO_2_ experiments (comprising the four models that conducted these: GFDL_CM2.1, HadCM3B_M2.1aN, HadCM3BL_M2.1aN, and MIROC4m), PI (top row) and early Eocene (bottom row); (b) higher‐level sample of CO_2_ experiments (comprising the two models that conducted these: CESM1.2_CAM5 and GFDL_CM2.1), PI (top row) and early Eocene (bottom row). Note that the PI panels are identical in each sample because they contain the same models, but are simply replicated here for ease of comparison.

**Figure 9:**
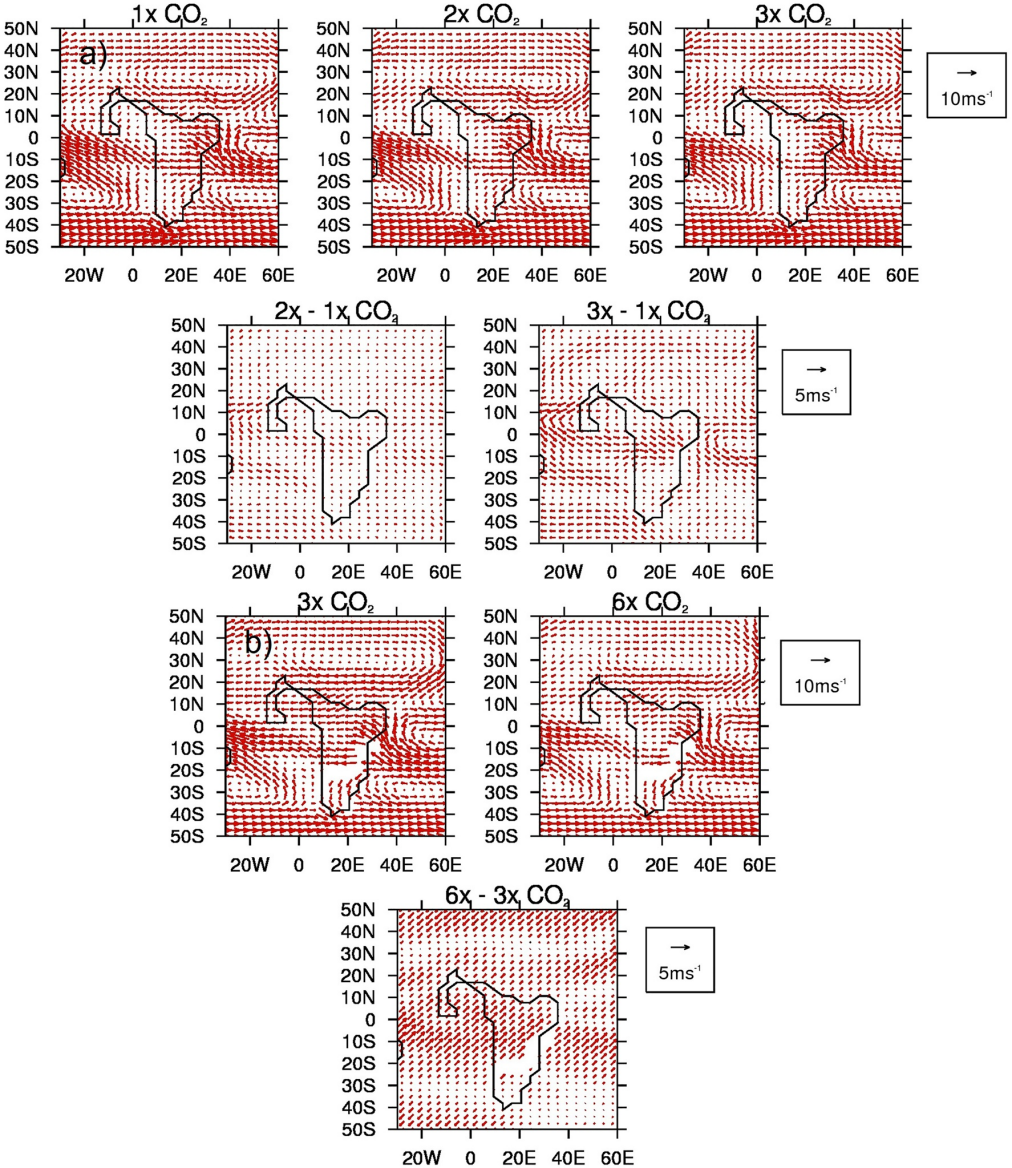
Same as Figure 5 but for June–August (JJA) 850 mb wind.

The MME absolute 1.5 m surface air temperature (SAT) and anomalies for the lower‐ and higher‐level sample of CO_2_ experiments are shown in Figure 7. In line with general understanding, there is a clear increase in absolute SAT, everywhere, as the CO_2_ increases, with the largest signal (of up to 40°C in the 3x experiment) occurring over the main landmass of central and northern Africa (Figure 7a, top row). This is more obvious when the anomalies are considered, although the largest increases are occurring further south (Figure 7a, second row). This is even more pronounced in the higher‐level sample of CO_2_ experiments (Figure 7b), and in all experiments, the largest increase in SAT, either between the 3x and 1x experiments or the 6x and 3x the experiments, is occurring over southern Africa, away from the largest precipitation changes discussed above. Moreover, the largest increases in precipitation as CO_2_ increases (Figure 5) are shown over ocean regions, such as the equatorial Atlantic and off the coast of West early Eocene Africa, whereas the largest increases in SAT (Figure 7) are shown over the landmass. It is likely that these precipitation increases are connected to the warmer SSTs (see Section 3.2.3), or changes to the low‐level circulation (discussed below), rather than a direct response to the heating landmass. The precipitation‐evaporation (P‐E) balance (Figure 8) is positive over West Africa in all experiments regardless of sample, corresponding well with the region of increased precipitation (Figure 6), as does cloud cover which is also increasing with CO_2_ over these regions (not shown). Further south, over the Atlantic, the balance is negative implying increased evaporation corresponding to the increased oceanic SAT. Concerning low‐level circulation, as shown by 850 mb vector winds (Figure 9), when the anomalies are considered (and in particular the 3x vs. 1x), there is a small (of up to 5 ms^−1^) increase in northerly and westerly winds (i.e., clockwise flow) in the equatorial Atlantic north of the Equator (Figure 9a, second row). However, in the higher‐level CO_2_ sample (and in particular the anomalies of 6x vs. 3x, Figure 9b, second row), this increase in anticyclonic flow is less evident and is instead replaced by a widespread area of increased southwesterly flow across most of the equatorial Atlantic and central Africa. For SAT, P‐E and 850 vector winds from each individual model, rather than the MME, see the Supporting Information (Figures S4a–S4c in Supporting Information S1, respectively); here, similar to Figure 4, there is no obvious linear change in either P‐E or low‐level circulation as CO_2_ increases, but a clear increase in SAT from all models, in line with current understanding (Figure S4a in Supporting Information S1).

Both the region of enhanced precipitation over West Africa, and the region of drying in the equatorial Atlantic around 10°N, may be explained by these low‐level circulation changes. Up to 3x that of the PI CO_2_, clockwise low‐level circulation increases with CO_2_, drawing in more moisture from the equatorial Atlantic and causing a relative drying further north, hence the appearance of a southward displacement of the Atlantic ITCZ. At higher levels of CO_2_, however, where increases in West African precipitation are shown but the region of drying around 10°N is not, the increased clockwise low‐level circulation is replaced by increased south‐westerly flow; here, therefore, precipitation is being enhanced by more moisture being drawn in by this south‐westerly flow from the warm South Atlantic.

### 3.4 DeepMIP Models' Eocene Simulations vs. Proxy Data

In this final section, the focus is on comparing precipitation from selected DeepMIP early Eocene simulations (using the MME from the same two samples as discussed above) with newly available precipitation reconstructions (described in Section 2.3.2). Before the results are presented, however, several sources of uncertainty in the proxies and models must be noted, aside from analytical uncertainty that is expressed in the reconstructed confidence intervals. First, the fossil plant assemblages analyzed here have broad age constraints. Paleofloral assemblages may capture a snapshot within those age constraints that deviated climatically from the average climatic conditions of a specific age that the model was calibrated on. In addition, fossil plant assemblages tend to preserve better in wetter climates, with drier climates lacking the water bodies needed to preserve plant fossils. Second, the DeepMIP models are calibrated on atmospheric CO_2_ proxy reconstructions to cover the uncertainty of the entire Eocene; the lower CO_2_ levels may be more representative of the late Eocene, but that was not the purpose or interpretation when it came to deciding the experiments. Independent proxies within those ages produce widely variable atmospheric CO_2_ reconstructions (e.g., Rae et al., 2021), with <500 ppmv from some paleosol and stomatal reconstructions (Beerling et al., 2009; Hyland et al., 2013) to >2,000 ppmv from boron isotopes and alkenone *δ* ^13^C (e.g., Anagnostou et al., 2020; Bijl et al., 2010). It should be noted, however, that there is high uncertainty in these reconstructions; see Hollis et al. (2019) for a full discussion. For example, based on a variety of reconstructions compiled as part of the Paleo‐CO_2_ project (including phytoplankton, boron proxies, leaf gas exchange, liverworts, and nahcolite), atmospheric CO_2_ during 55–50 Ma ranges from 500 to 2,000 ppmv (Anagnostou et al., 2020; Hollis et al., 2019; Westerhold et al., 2020). Potentially, these differences in reconstructed atmospheric CO_2_ reflect transient climate states (e.g., Reichgelt et al., 2016), but regardless, the disagreement between proxy reconstructions makes it problematic to associate a single atmospheric CO_2_ level for model‐data comparison (Hollis et al., 2019). Lastly, a major source of uncertainty is the paucity of proxy data across Africa; as mentioned above, even today there is a lack of long‐term climate data over much of Africa, and the same is true for paleofloras. This sparsity, therefore, is likely responsible for some of the results discussed below, and this is why some of the following results are necessarily partly speculative.

**Figure 10:**
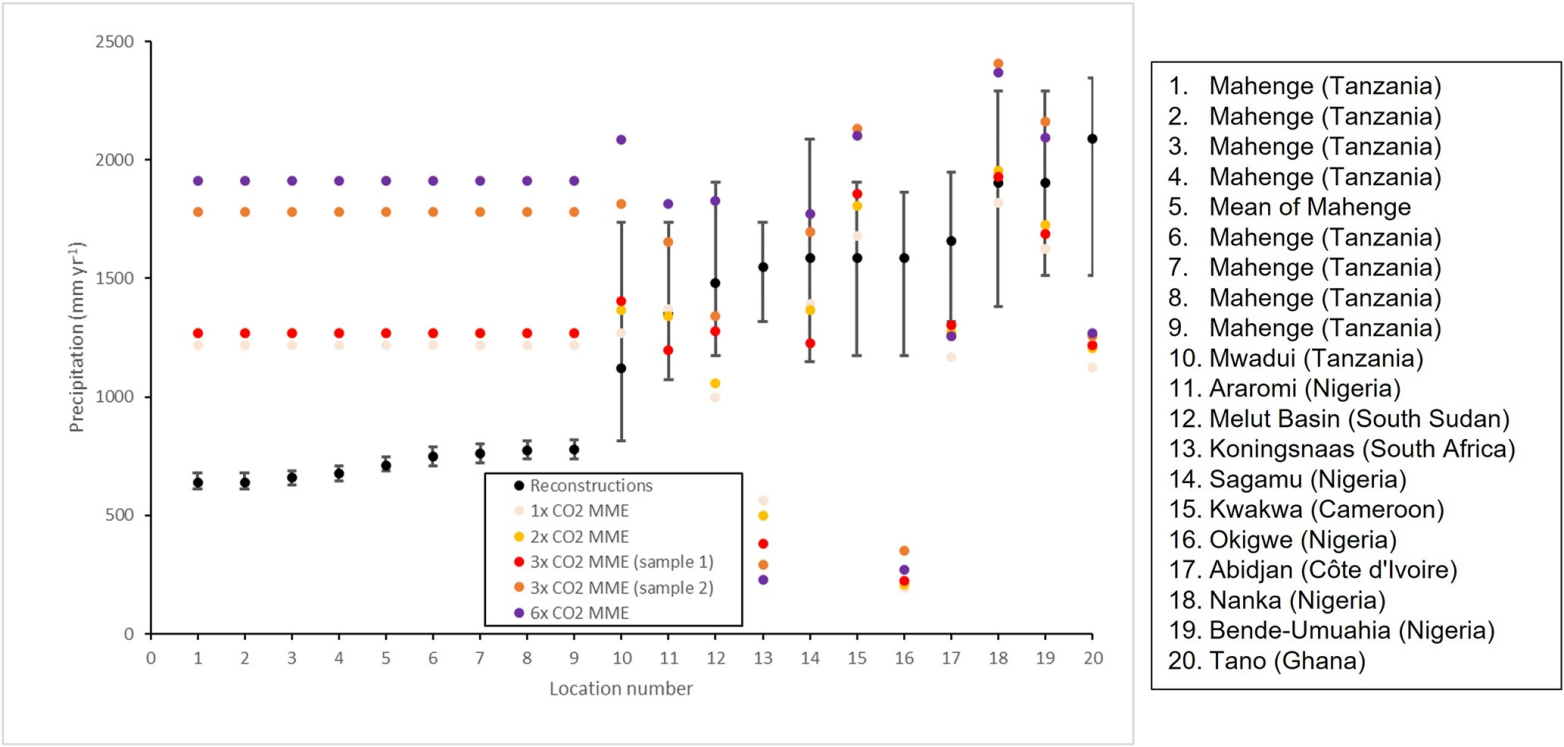
Annual mean precipitation from reconstructions (black) and CO_2_ experiments multi‐model ensemble mean (MME, colors) at each individual location. Uncertainty in reconstructions is measured by 95% confidence interval for all sites except Mahenge, where they show +/−1 standard deviation. Locations have been ordered according to the reconstructions' values, lowest to highest. Note that locations 1–4 and 6–8 are all in the same location, but from different stages during the Lutetian (∼41–47 Ma), and so have been re‐sampled and averaged into one overall mean (location 5).

With these caveats in mind, MME MAP at each of the individual locations is shown in Figure 10, ordered according to the reconstructions' values, including uncertainty estimates for the reconstructions (as measured by ±1 standard deviation for the locations in Mahenge, Tanzania and the 95% confidence interval for the other 11 locations; see Table 2 for details). The approximate geographical locations can be seen in the Supporting Information (Figure S5 in Supporting Information S1). First it is worth noting that for the majority of reconstructions, uncertainty is high, with a range of up to +/−1,000 mm yr^−1^ at some of the locations such as Mwadui, Tanzania (Figure 10). Second, whether or not the CO_2_ experiments over‐ or underestimate MAP appears to depend heavily on geographical location, with none of the CO_2_ experiments (not even the 6x experiment) reproducing the precipitation amounts of the proxy reconstructions in some locations, such as Koningsnaas, South Africa, Okigwe, Nigeria or Tano, Ghana (Figure 10). Elsewhere, the simulations lie within the uncertainty range of the reconstructions (such as Sagamu or Bende‐Umuahia, both in Nigeria), and yet in other places (such as across Kwakwa, Cameroon, and all of the locations at Mahenge, Tanzania) all of the simulations are too wet, by between ∼760 and 1,040 mm yr^−1^ depending on location and CO_2_ experiment (Figure 10).

**Figure 11:**
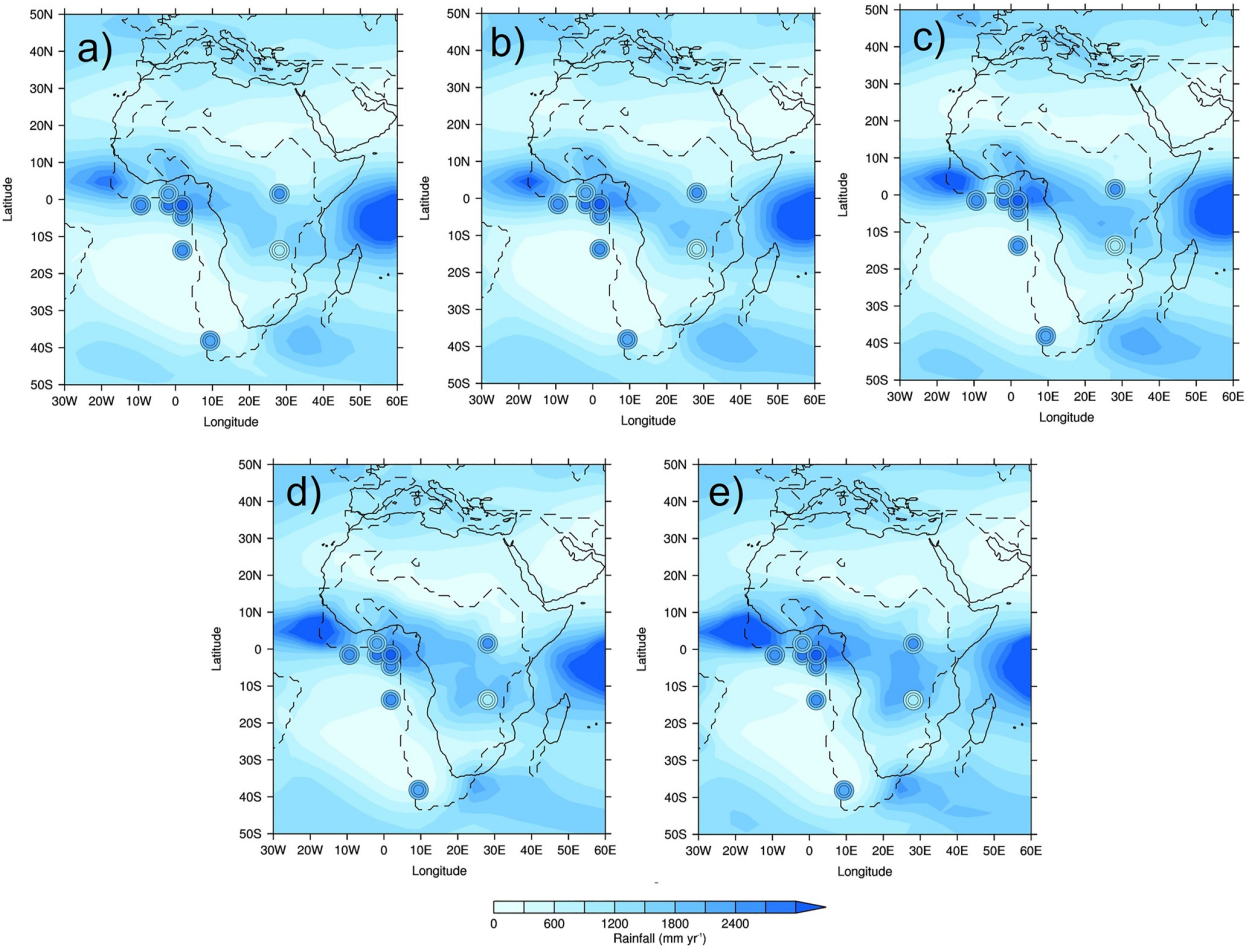
Annual mean precipitation from reconstructions (circles) and CO_2_ experiments multi‐model ensemble mean (MME, background gridded data): (a) 1x; (b) 2x; (c) 3x (lower‐level CO_2_ sample); (d) 3x (higher‐level CO_2_ sample); (e) 6x. Concentric circles show 95% confidence interval for all sites except Mahenge, where they show +/−1 standard deviation: outer circle = lower range (or −1 standard deviation), middle circle = average (or, for Mahenge, mode) and inner circle = upper range (or +1 standard deviation). Reconstructions have been rotated forwards to where they are in the PI. Solid lines show the PI mask and dashed lines show the early Eocene mask. Note that, using the common spatial resolution of the MME, 3 reconstructions are all in the same location in West Africa (even though they are in different locations in reality); here, therefore, only the top‐most reconstruction is shown.

Spatially, MME MAP is shown in Figure 11 (see Figure S6 in Supporting Information S1 for each individual model), showing the uncertainty estimates as concentric circles. As already discussed, the simulations' precipitation is clearly too high or too low compared to proxy reconstructions in different parts of Africa. Qualitatively, in very general terms all of the CO_2_ experiments are showing wetter conditions over Western early Eocene Africa (relative to elsewhere), agreeing with Figure 10 where in many of these locations the models are either within, or at the higher end of, the reconstructions' uncertainty ranges (Figure 11). Importantly, simulated precipitation over West Africa appears to be increasing as the CO_2_ concentration increases and, in particular for the 6x experiment (Figure 11e), in this region simulated precipitation exceeds even the upper range of uncertainty of the reconstructions.

Quantitatively, the root mean squared error (RMSE) between each model (as well as the MME) and the reconstructions at every location is shown in Table 3 and, similar to the anomalies from each model as discussed above, there is no clear relationship between changing CO_2_ and a better match to the reconstructions. Most models suggest a better fit to the reconstructions at lower levels of CO_2_, such as CESM1.2_CAM5 where there is a general increase in RMSE as the CO_2_ increases; however, this is not the case for every model, with for example, GFDL_CM2.1 showing a better fit with reconstructions at 2x and 4x CO_2_, rather than higher or lower levels (Table 3). For many of the models and the MME, the 3x CO_2_ experiments are showing the least fit with reconstructions. The MME, from the lower‐level (but not in the higher‐level) CO_2_ sample, agrees with this conclusion that lower CO_2_ is giving a slightly better match to the reconstructions, with RMSE values of 758 mm yr^−1^, 831 mm yr^−1^, 1,385 mm yr^−1^, 889 mm yr^−1^ and 839 mm yr^−1^ for the 1x, 2x, 3x (lower‐level CO_2_ sample), 3x (higher‐level CO_2_ sample) and 6x experiments, respectively (Table 3).

**Table 3:** Root Mean Squared Error (RMSE) for Mean Annual Precipitation (MAP) Between Each Model (and Multi‐Model Ensemble Mean, MME, Using Both Samples) and Reconstructions, for Each CO_2_ Experiment

	1x CO_2_	1.5x CO_2_	2x CO_2_	3x CO_2_	4x CO_2_	6x CO_2_	9x CO_2_
CESM1.2_CAM5	681			750		704	822
COSMOS‐landveg_r2413	699			1,424	713		
GFDL_CM2.1	803		762	1,027	786	975	
HadCM3B_M2.1aN	796		884	1,988			
HadCM3BL_M2.1aN	816		1,018	1,742			
INM‐CM4‐8						966	
IPSLCM5A2		744		669			
MIROC4m	614		662	785			
NorESM1_F			1,149		1,522		
MME (lower‐level CO_2_ sample)	758		831	1,385			
MME (higher‐level CO_2_ sample)				889		839	

## 4 Discussion and Conclusions

This study has investigated African precipitation during the early Eocene, as simulated by the DeepMIP models. This study is novel, because it investigates the relatively little‐studied subject of African hydroclimate during the early Eocene. The results of this study have been divided into four separate sections, corresponding to the four questions posed in Section 1. First, in Section 3.1 the DeepMIP models' PI simulations have been compared to satellite‐derived estimates of precipitation, to ascertain how well the models are able to reproduce African precipitation under “modern” conditions (please see Section 2.3.1 for a discussion of the caveat that here the term “modern” is actually a combination of both pre‐industrial and 20th‐21st century). Second, in Section 3.2 the DeepMIP models' early Eocene simulations have been compared to both the PI simulations and each other, to investigate the impact of CO_2_ components (i.e., increasing CO_2_) and non‐CO_2_ components (i.e., other boundary condition changes, such as to the LSM) on African precipitation. Third, in Section 3.3 the CO_2_ driven response has been investigated further by looking at a number of dynamic and thermodynamic fields simulated by the models, to ascertain possible physical mechanisms behind the observed precipitation response. Lastly, in Section 3.4 the DeepMIP models' early Eocene simulations have been compared to newly available proxy data, to indicate how well the models agree with current best precipitation estimates from the Eocene.

The comparison between the DeepMIP PI simulations and modern observations (from TAMSAT) suggest that individual models are both underestimating or overestimating the spatial patterns of African precipitation; this is consistent with Monerie et al. (2020), who analyzed a number of historical simulations from both CMIP5 and CMIP6 and found that the models' ability to reproduce observations was first model dependent and second geographically dependent, with many models underestimating precipitation over the Sahel and overestimating it over the Guinea coast and tropical Atlantic. However, here the MME is reducing these biases and is showing the best agreement with TAMSAT in terms of precipitation spatial patterns, highlighting the utility of the MME as a best estimate of the actual precipitation. This has been found elsewhere, such as by Ayugi et al. (2021) who looked at East African precipitation in both CMIP5 and CMIP6 models and again found a better performance of the MME relative to individual models, due to systematic errors in individual models being canceled out. Moreover, Rougier et al. (2013) show that it is actually a statistical property of this type of analysis that the ensemble mean will always provide the best match to the data for example, have the lowest RMSE. It should be noted, however, that a potential caveat of using the MME is that although it eliminates extreme biases, the same models are then being used to run the Eocene simulations, for which the correct (i.e., true) precipitation is less well known and based only on paleodata, which themselves have uncertainties. Therefore, even using the MME may be propagating its own unknown errors. Concerning the latitudinal extent and seasonal timings of African precipitation, most models show a much wider (latitudinally) West African rain belt compared to TAMSAT and are not reproducing the rapid drop‐off in precipitation near the Equator or north of 15°N. This is somewhat in contrast to Monerie et al. (2020), who noted that the majority of CMIP5 and CMIP6 models did not have the monsoon extending far enough to the north and were instead showing a southward displacement of precipitation maxima, relative to observations; however, that particular study used the models' historical simulations (as well as a different MME), not pre‐industrial as shown here, which may explain the discrepancy. Outside of JJA most models are too wet, but within JJA the results suggest that the drier models (i.e., those underestimating African precipitation) are closer to modern observations than those that are too wet (i.e., overestimating African precipitation).

The comparison between the DeepMIP early Eocene simulations and the PI suggests that, when all individual models are considered separately, there is no obvious wetting or drying trend (relative to the PI) as the CO_2_ increases. This is another reason to focus on the MME, which allows easier interpretation as the large model spread is removed. Concerning the non‐CO_2_ component of precipitation change (i.e., the impact of other boundary conditions when CO_2_ is kept at PI levels), the results suggest that changes to the LSM may be responsible for the increases in precipitation (relative to the PI) to the north of early Eocene Africa and the western Indian Ocean, given that these are “newly exposed” regions of ocean in the early Eocene, thereby providing a larger moisture source. In contrast, it is likely that changes in orographic heights are responsible for the region of drying (relative to the PI) over equatorial early Eocene Africa, where early Eocene Africa is considerably lower (up to 400 m) than in the PI (up to 1,000 m). When the early Eocene precipitation is rotated forwards in time to where it is in the PI, a similar pattern is shown but is more pronounced, and suggests a northward displacement of the primary rain belt (relative to today), which is consistent with previous work (e.g., Carmichael et al., 2016). Concerning the CO_2_ component of precipitation change, at the lower levels of increased CO_2_ (such as 2x and 3x that of the PI) precipitation over the equatorial Atlantic and West Africa appears to be increasing in response to rising CO_2_, with the concomitant decrease in precipitation north of the equator suggesting a possible displacement of the Atlantic ITCZ toward the south. This therefore suggests that the boundary condition changes imposed for the Eocene are resulting in a northward displacement of the primary rain belt, but increasing CO_2_ (with the same boundary conditions) is resulting in a southward displacement of the primary rain belt. At even higher levels of CO_2_ (such as 6x that of the PI), precipitation over West Africa is more enhanced relative to the lower levels, but the region of drying is less evident. The enhancement of Northern Hemisphere summer West African precipitation at the highest levels of CO_2_ is again consistent with previous work, such as that of Carmichael et al. (2016) who showed a generally more intense hydrological cycle at higher CO_2_ levels and that of Carmichael et al. (2018) who demonstrated an increase in precipitation extremes over tropical Africa at higher CO_2_ levels.

Consistent with Carmichael et al. (2016), the precipitation increases over West Africa as CO_2_ concentrations rise are associated with increased SAT, a strongly positive the P‐E balance and cloud cover increases and, concerning temperature, as such are consistent with the idea that a generally warmer world results in a generally wetter world; the “wet‐gets‐wetter and dry‐gets‐drier” hypothesis (e.g., Held & Soden, 2006). However, the largest increases in SAT shown here are over southern Africa, not where the largest precipitation increases are seen, suggesting factors other than a generally warming world (i.e., dynamical changes) are responsible for the localized precipitation response (see Section 3.3). A caveat to mention here is that, because the DeepMIP simulations use prescribed vegetation rather than interactive, there is no impact on the vegetation types or distribution of these enhanced SATs or precipitation, therefore it is not possible to say whether any enhanced precipitation would be enough to support a certain type of vegetation in the presence of extreme temperatures. Whilst it is likely that the impacts of elevated temperatures and precipitation (whether combined or individually) would be substantial on plant physiology, it is beyond the scope of this study to test this. Sensitivity studies, using interactive vegetation, are currently underway to address these questions.

Lastly, the results from the model‐data comparison suggests that whether the early Eocene simulations (regardless of CO_2_ experiment) over‐ or underestimate African precipitation is highly geographically dependent, with some of the CO_2_ experiments at some of the locations lying within the uncertainty range of the reconstructions but others being too wet or too dry. There is some suggestion of a latitudinal relationship, with the simulations overestimating precipitation near the Equator and underestimating precipitation in high latitude regions, such as South Africa; this latter point is consistent with the findings of Carmichael et al. (2016). Whether the models are considered independently or whether the MME is used, the results suggest a marginally better fit with the reconstructions at lower levels of CO_2_, and this is in contrast (indirectly) to the findings of Carmichael et al. (2016) who suggested the warmest models in the regions of increased precipitation best matched the data; it should be noted, however, that this was a global study. There is no evidence for this here, and indeed the finding of a better match at lower levels of CO_2_ is in contrast to that of Reichgelt et al. (2022) who focused on Australia and found that the higher, 6x CO_2_ experiment was the best match to reconstructions. However, given the uncertainties associated with both the reconstructions (discussed above) and the boundary conditions used to force the models, it is difficult to draw firm conclusions from a model‐data comparison of this type. Moreover, a particularly big problem is that, despite the newly compiled reconstructions presented here, there is still a lack of data across Africa, hindering any firm conclusions. The uncertainties discussed above are likely contributing to the lack of consistency presented in some of these model‐data comparisons, such as the MME showing better agreement with the reconstructions at lower and higher levels of CO_2_, but not in between (e.g., the 3x simulation), but this is, at present and given the data sparsity, unavoidable.

In conclusion, therefore, this study has shown that the DeepMIP models are able to approximately reproduce the modern African precipitation and, in response to rising CO_2_, suggest an enhancement of precipitation in this region associated with increasing temperatures and changes to low‐level circulation. At very high levels of CO_2_ the models may be too wet, relative to proxy reconstructions. However, this might be because the NLR proxy approach has difficulty generating MAP values above modern, or connected to the relatively few early Eocene‐aged data points within the reconstructions (meaning some of the comparisons here were necessarily made with data from the middle or late Eocene). Using the MME provides the clearest suggestion of this, but the large amount of model spread means that when individual models are considered, either relative to their corresponding PI simulations or reconstructions, no clear relationship is shown.

## Conflict of Interest

The authors declare no conflicts of interest relevant to this study.

## Supporting information

Supporting Information S1

## Data Availability

TAMSAT data are publicly available to download at https://www.tamsat.org.uk/; please see Maidment et al. (2014, 2017) and Tarnavsky et al. (2014). The paleobotanical precipitation estimates compiled here are available as a spreadsheet, available to download at Williams (2022). The DeepMIP PI and Eocene simulations are available by following the instructions at https://www.deepmip.org/data-eocene/; please see Hollis et al. (2019).
