# Supplementary material for: African Hydroclimate During the Early Eocene From the DeepMIP Simulations
Source: Paleoceanogr Paleoclimatol. 2022 May 16;37(5):e2022PA004419. doi: 10.1029/2022PA004419 (PMC9321955; doi:10.1029/2022PA004419)
Supplement: Supplementary file 1 — Supporting Information S1 [file PALO-37-0-s001.docx]

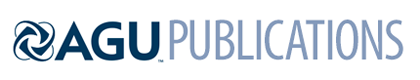


*Paleooceanography and Paleoclimatology*

Supporting Information for

**African hydroclimate during the early Eocene from the DeepMIP simulations**

Charles J. R. Williams^1,2^, Daniel J. Lunt^1^, Ulrich Salzmann^3^, Tammo Reichgelt^4^, Gordon N. Inglis^5^, David R. Greenwood6, Wing-Le Chan^7^, Ayako Abe-Ouchi^7^, Yannick Donnadieu^8^, David K. Hutchinson^9,10^, Agatha M. de Boer^9^, Jean-Baptiste Ladant^8^, Polina A. Morozova^11^, Igor Niezgodzki^12,13^, Gregor Knorr^13^, Sebastian Steinig^1^, Zhongshi Zhang^14^, Jiang Zhu^15^, Matthew Huber^16^, Bette L. Otto-Bliesner^15^

^1^School of Geographical Sciences, University of Bristol, UK

^2^NCAS / Department of Meteorology, University of Reading, UK

^3^Geography and Environmental Sciences, Northumbria University, UK

^4^Department of Geosciences, University of Connecticut, USA

^5^School of Ocean and Earth Science, University of Southampton, UK

^6^Department of Biology, Brandon University, Canada

^7^Atmosphere and Ocean Research Institute, The University of Tokyo, Japan

^8^Laboratoire des Sciences du Climat et de l’Environnement, France

^9^Department of Geological Sciences, Stockholm University, Sweden

^10^Climate Change Research Centre, University of New South Wales, Australia

^11^Institute of Geography, Russian Academy of Sciences, Russia

^12^Institute of Geological Sciences, Polish Academy of Sciences, Poland

^13^Alfred Wegener Institute for Polar and Marine Research, Germany

^14^Bjerknes Centre for Climate Research, University of Bergen, Norway

^15^Climate and Global Dynamics Laboratory, National Center for Atmospheric Research, USA

16Department of Earth, Atmospheric and Planetary Sciences, Purdue University, USA

**Contents of this file**

Figures S1 to S6

Tables S1 to S2

**Introduction**

The figures and tables included here provide supporting information for the above manuscript.


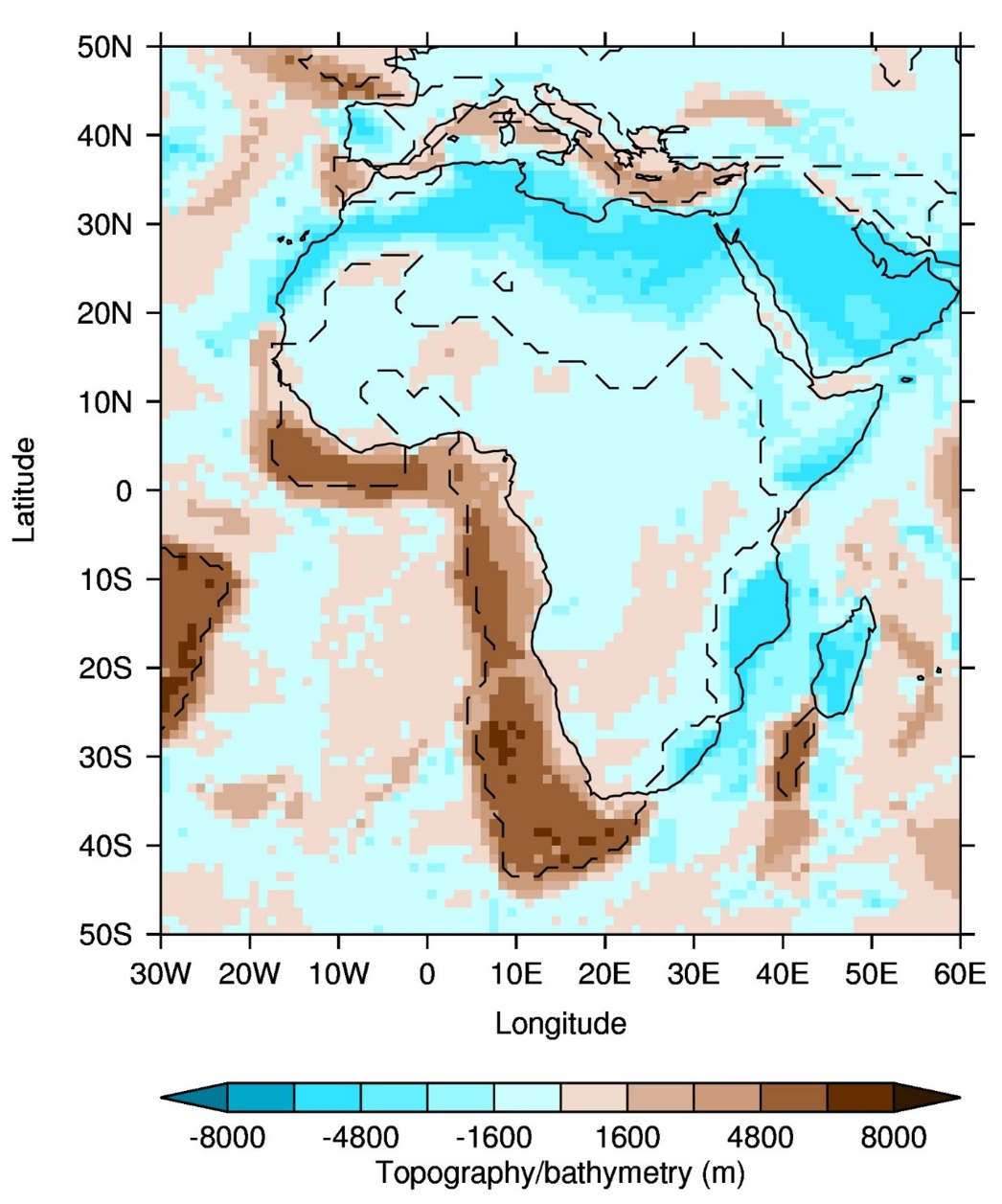


Figure S1. Topography/bathymetry changes in DeepMIP simulations. Solid line shows PI land sea mask, dashed line shows early Eocene land sea mask

***
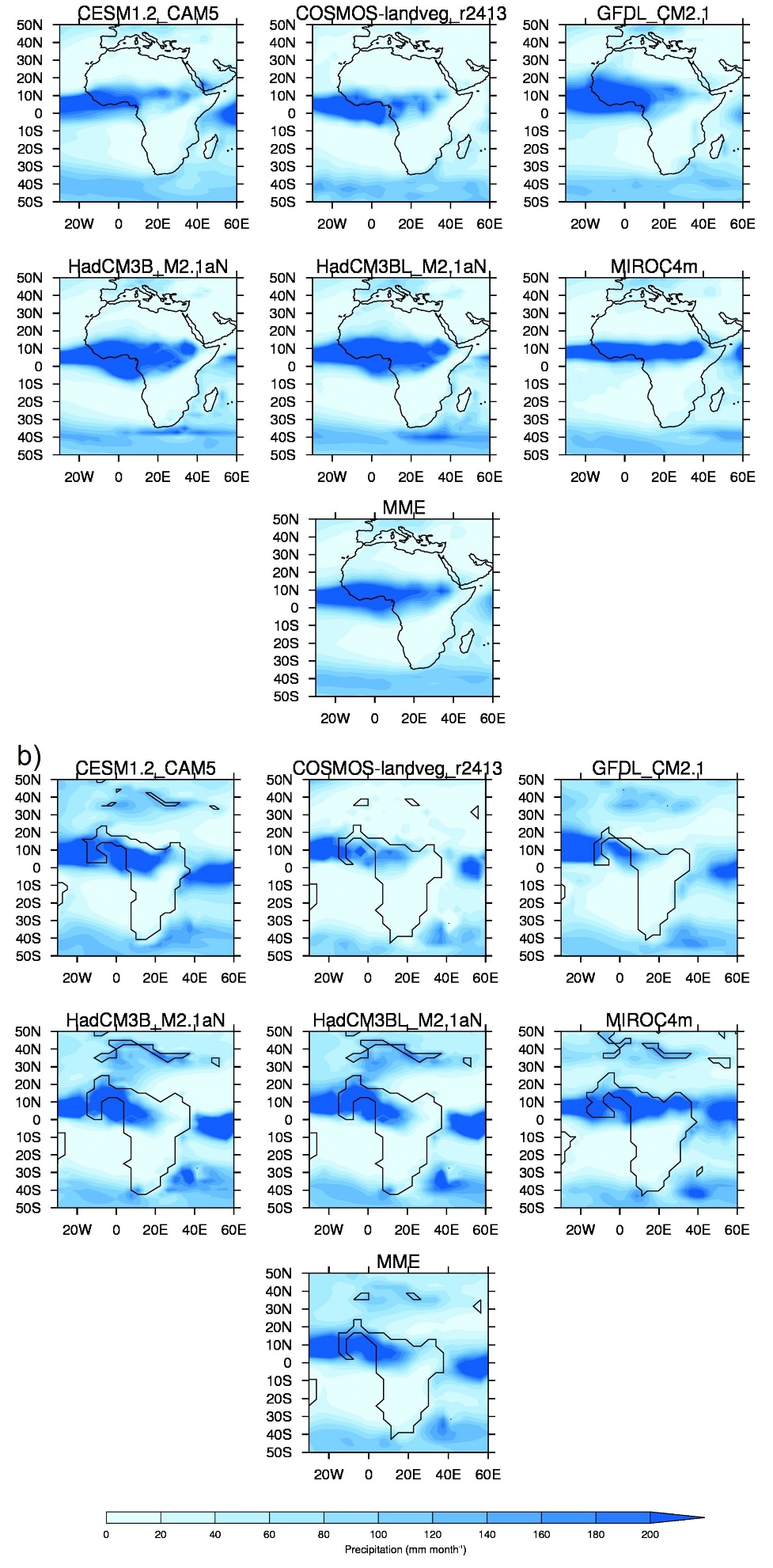
***

**Figure S2**. JJA precipitation from each model conducting the 1x CO2 experiment, as well as multi-model ensemble mean (MME): a) PI; b) early Eocene


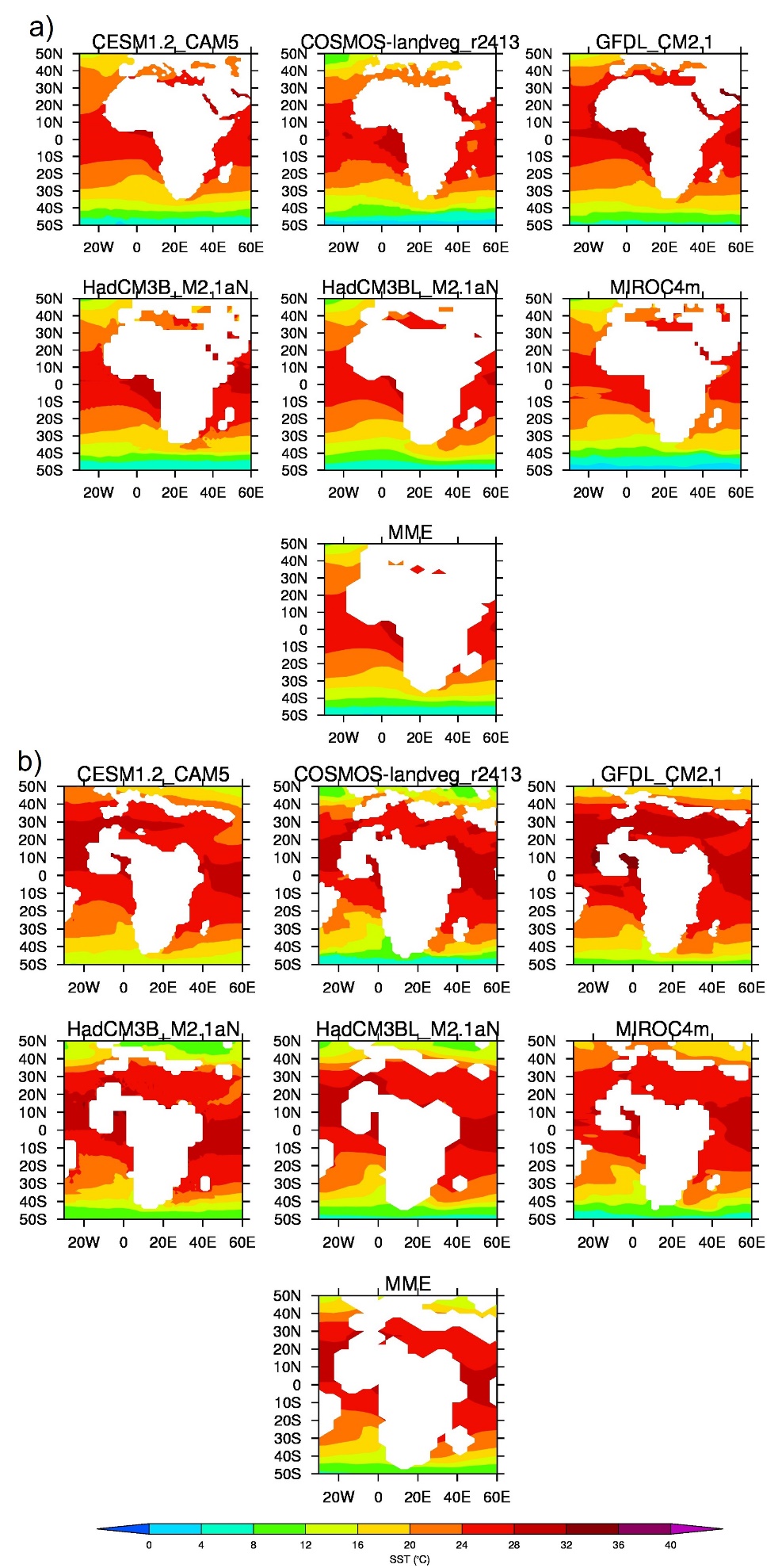


**Figure S3**. JJA sea surface temperature (SST) climatologies from each model conducting the 1x CO2 experiment, as well as multi-model ensemble mean (MME): a) PI; b) early Eocene


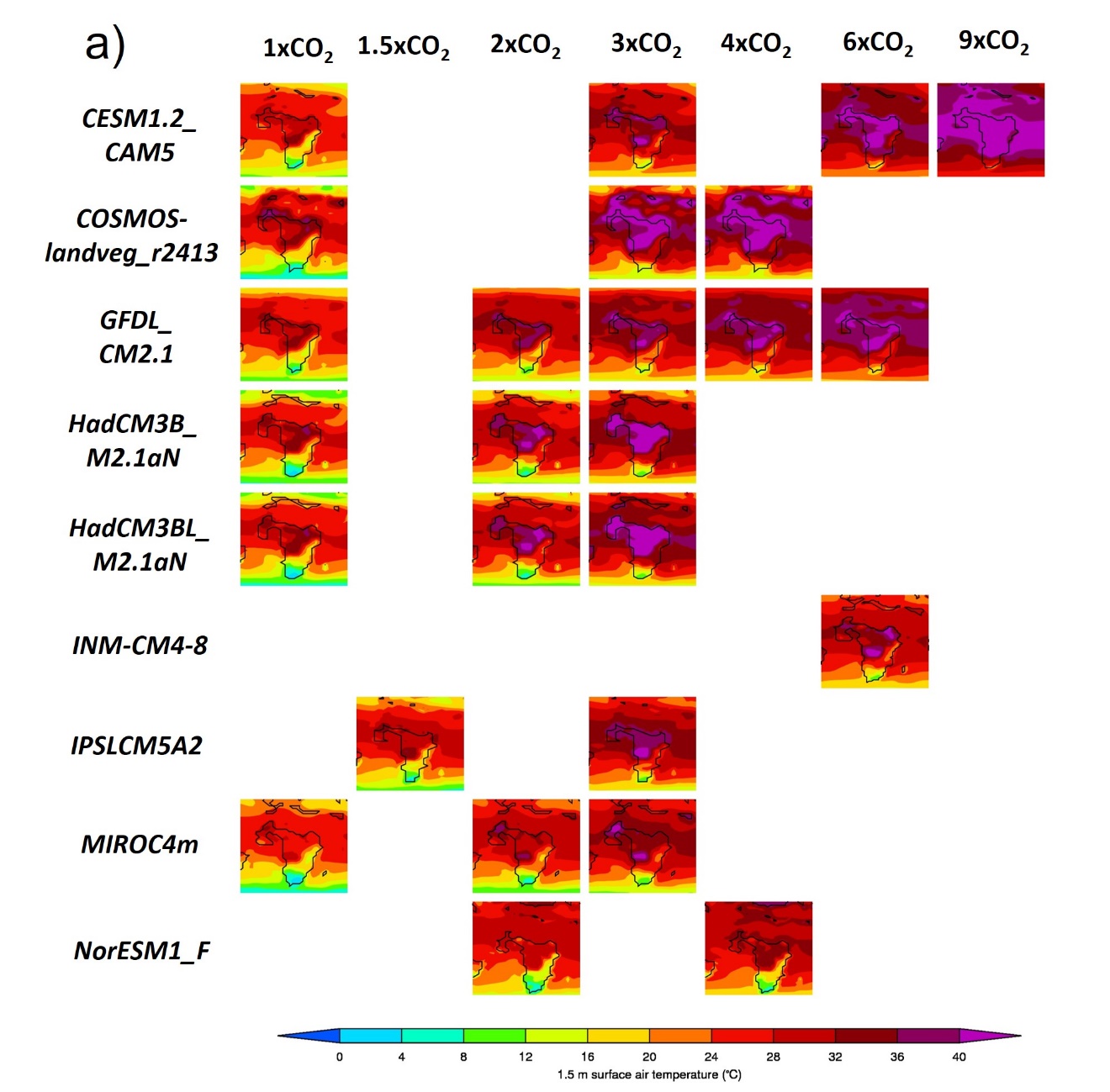


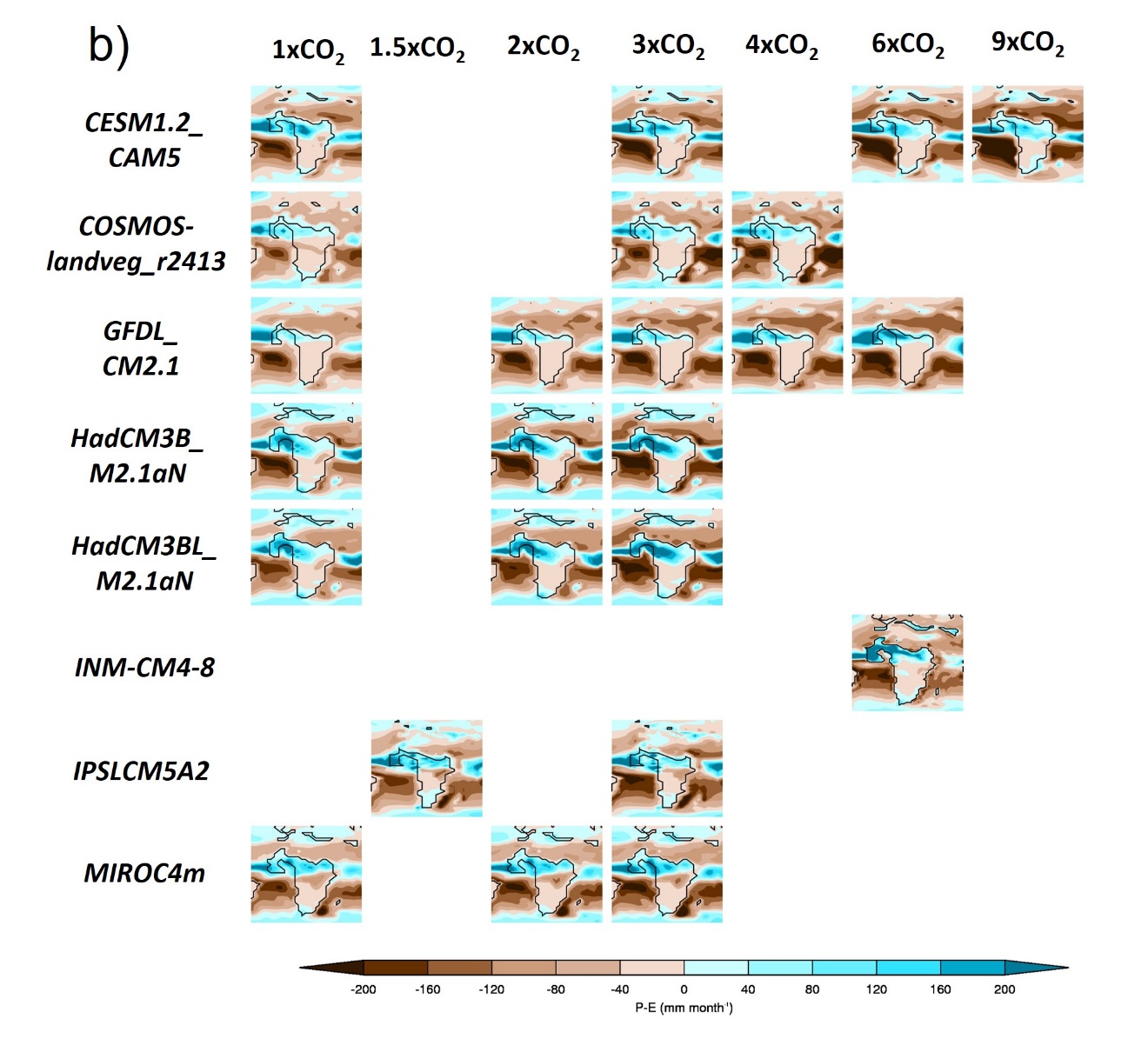


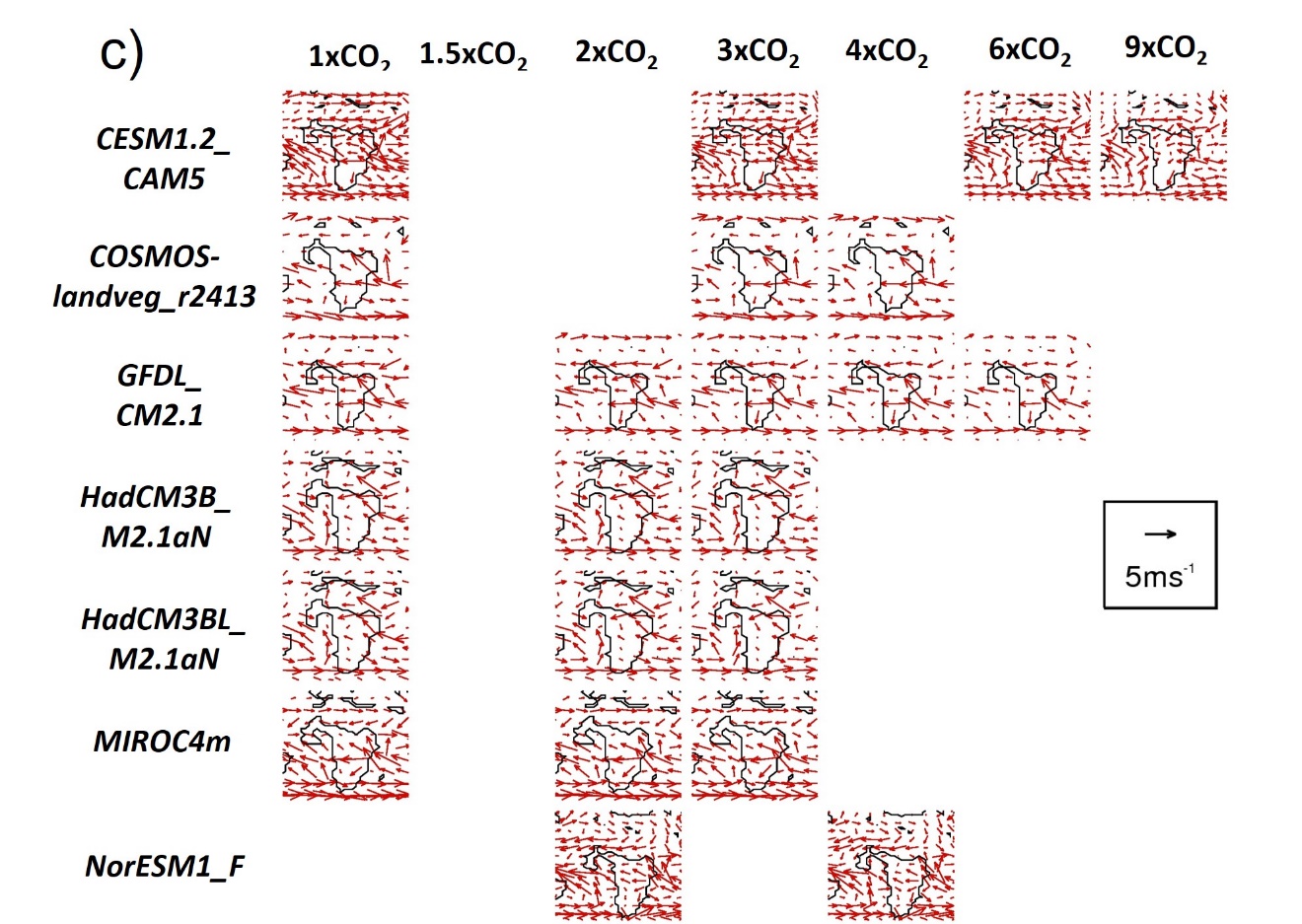


**Figure S4**. JJA climatologies for each CO2 simulation from each model: a) 1.5 m surface air temperature (SAT); b) JJA Precipitation - Evaporation (P-E); c) 850 mb vector winds. Note that in b), evaporation data are missing from NorESM1_F, hence its exclusion here, and likewise in c), wind data are missing from INM-CM4-and IPSLCM5A2, hence their exclusion here


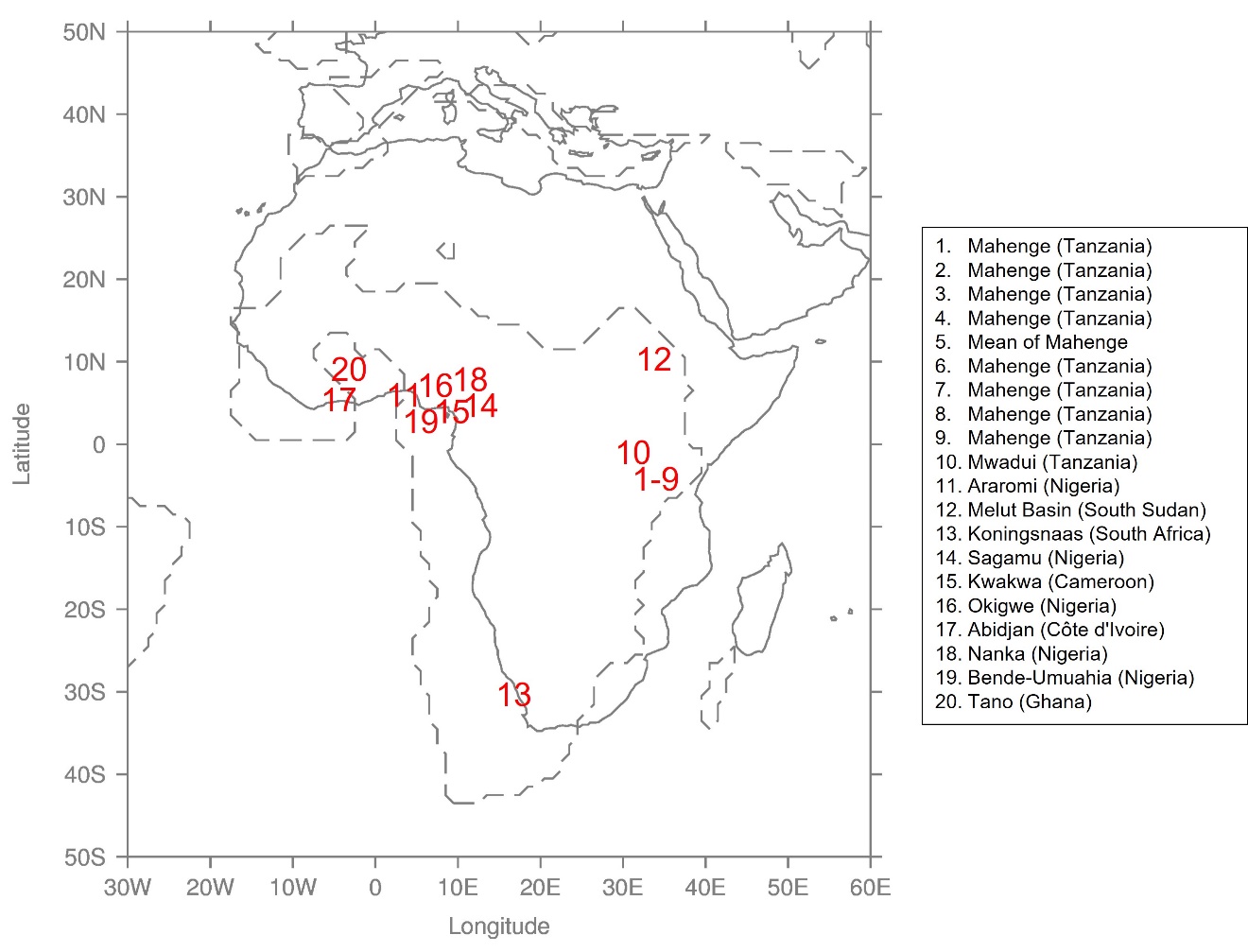


**Figure S5**. Approximate locations of reconstructions across Africa, ordered according to the reconstructions’ values, lowest to highest. Note that locations 1-9 are all in the same location, but from different stages during the Lutetian (~41-47 Ma), and so have been re-sampled and averaged into one overall mean (location 5). Reconstructions have been rotated forwards to where they are in the PI. Solid lines show the PI mask and dashed lines show the early Eocene mask.


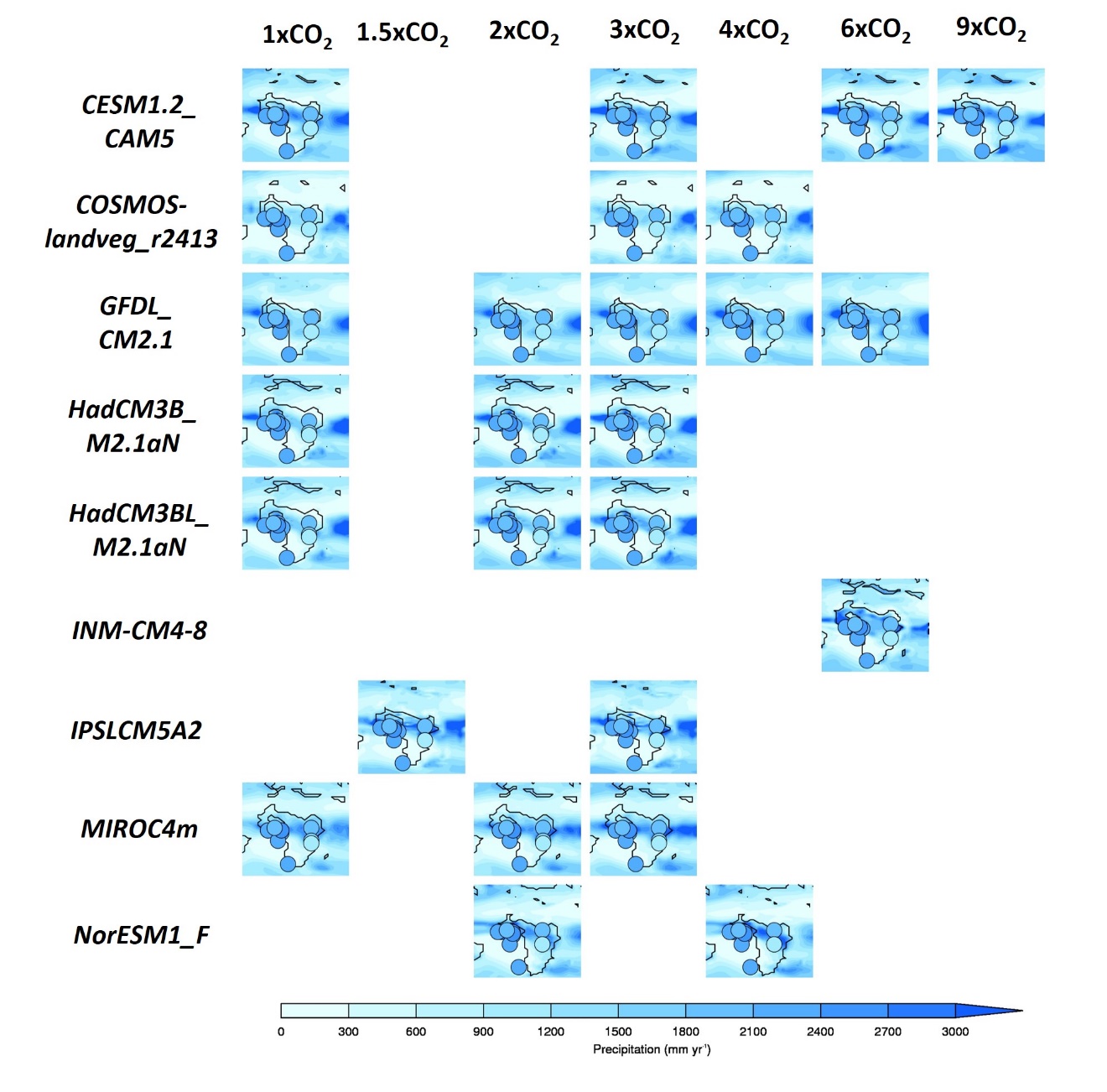


**Figure S6**. Annual mean precipitation from reconstructions (circles) and CO2 experiments (background gridded data) for each individual model. Locations in Mahenge, Tanzania are in the same place, so have been averaged. Reconstructions have been rotated forwards to where they are in the PI. Note that, for the majority of models, the spatial resolution is such that 3 reconstructions are all in the same location in West Africa (even though they are in different locations in reality); here, therefore, only the top-most reconstruction is shown

| **Site** | **Lat (°N)** | **Lon (°E)** | **Age** | **Reference** | **Taxa** |
| --- | --- | --- | --- | --- | --- |
| Koningsnaas | -30.2 | 17.3 | Paleocene-Eocene | de Villiers (1997) | *Pteris*, Cyatheaceae, *Acrostichum,* Matoniaceae Gleicheniaceae, *Lygodium,* Osmundaceae, Polypodiaceae, Podocarpaceae, *Gevuina*/*Hicksbeachia,* Proteaceae, Apocynaceae, Sterculioideae, Arecaceae, Liliaceae, Asteraceae, Buxaceae, *Gunnera* Polygonaceae, Sapotaceae, Polygalaceae, Cupanieae, *Araucaria/Agathis* |
| Shagamu | 6.7 | 3.7 | Paleocene-Eocene | Adeonipekun *et al*. (2012) | Araceae, Arecaceae, *Adenanthera*, *Croton,* Nypoideae, *Cleistopholis*, Lepidocaryeae, Lecythidaceae, *Ludwigia,* Cupanieae |
| Melut Basin | 10 | 33 | Ypresian | Eisawi & Schrank (2008) | Cyatheaceae, *Ceratopteris,* Polypodiaceae/Blechnaceae, *Symphonia, Caesalpinia,* Bombacoideae, Lepidocaryeae, *Lygodium,* Osmundaceae, Poaceae, *Crudia,* Polypodiaceae, *Ludwigia*, Apocynaceae |
| Kwakwa | 4.5 | 9.1 | Paleocene-Eocene | Salard-Cheboldaeff (1979) | Nypoideae, Arecaceae, Ulmaceae, *Amanoa, Crudia*, Poaceae, Lepidocaryeae, *Calystegia, Alchornea, Anacolosa,* Bombacoideae |
| Mwadui | -3.9 | 33.5 | Ypresian | Cantrill *et al*. (2013) | *Cynometra*, Simaroubaceae, Liliaceae, Cyatheaceae, Cyperaceae, Schizaeaceae, Proteaceae |
| Tano | 4.7 | -3 | Ypresian | Atta-Peters and Salami (2004) | *Amanoa*, Araceae, Bombacoideae, *Calamus*, Clusiaceae, *Ctenolophon*, Lepidocaryeae, *Monstera*/*Gonatopus*, Nypoideae, Proteaceae, *Stenochlaena* |
| Nanka | 6.12 | 7 | Ypresian | Okeke and Umeji (2016) | *Stenochlaena*, Arecaceae, *Calamus*, Lepidocaryeae, Iridaceae, Poaceae, Iriarteeae, Araceae, *Monstera*/*Gonatopus*, Bombacoideae, Nypoideae |
| Abidjan margin | 5 | -4.1 | Ypresian | Goha *et al.* (2016) | Cupressaceae, *Amanoa*, *Anemia*, Araceae, Arecaceae, Bombacoideae, Clusiaceae, *Ctenolophon*, Fabaceae, *Illicium*, Iridaceae, Lepidocaryeae, *Monstera*/*Gonatopus*, Nypoideae, Rhiozphoraceae, *Stenochlaena* |
| Okigwe | 5.82 | 7.34 | PETM | Uzodimma (2013) | Arecaceae, Proteaceae, *Pinanga*, *Nypa*, Cyatheaceae, Bombacaceae, *Ludwigia*, Ericaceae, *Geonoma*, Chenopodiaceae, *Ephedra*, *Ilex*, Meliaceae, *Anemia*, *Caesalpinia*, Poaceae, *Astrocaryum*, *Crudia*, Myrtaceae |
| Bende - Umuahia | 5.47 | 7.45 | Ypresian-Lutetian | Chiaghanam *et al*. (2017) | *Anemia,* Blechnaceae*, Stenochlaena,* *Amanoa,* Poaceae*, Nypa, Astrocaryum, Pinanga,* Bombacaceae*, Crudia, Ctenolophon,* Clusiaceae |
| Araromi | 7.7 | 3.5 | Ypresian-Lutetian | Salami (1984) | Arecaceae, *Pinanga*, Liliaceae, Cycadales, *Nypa*, *Nyssa*, Poaceae, Proteaceae, Smilax, *Lygodium*, Polypodiaceae/Blechnaceae |
| Mahenge | -4.79 | 34.26 | Lutetian | Jacobs and Herendeen (2004) | - |
| Mahenge | -4.79 | 34.26 | Lutetian | Jacobs and Herendeen (2004) | - |
| Mahenge | -4.79 | 34.26 | Lutetian | Jacobs and Herendeen (2004) | - |
| Mahenge | -4.79 | 34.26 | Lutetian | Jacobs and Herendeen (2004) | - |
| Mahenge | -4.79 | 34.26 | Lutetian | Jacobs and Herendeen (2004) | - |
| Mahenge | -4.79 | 34.26 | Lutetian | Jacobs and Herendeen (2004) | - |
| Mahenge | -4.79 | 34.26 | Lutetian | Kaiser et al. (2006) | - |
| Mahenge | -4.79 | 34.26 | Lutetian | Kaiser et al. (2006) | - |

Table S1. Paleocene-Eocene palaeobotanical records from Africa. Note that the eight sites at Mahenge are from slightly different time-slices within the Lutetian, and have therefore been averaged when making the model-data comparison. Moreover, these sites were generated by Leaf Area Analysis (LAA), not Nearest Living Relative (NLR) estimates, therefore do not have associated taxa. Note also that the exact ages of each site, and which best overlap with the DeepMIP simulations, is uncertain; the simulations represent ~50 Ma, whereas the Ypresian sites span ~47-56 Ma, the Lutetian sites span ~41-47 Ma and the Paleocene sites are generally ~56 Ma and earlier.

| **Plant group** | **GBIF DOI** | **MAP**  log mm | **WMP**  log mm | **DMP**  log mm | **PS** (CV) | **WQP**  log mm | **CQP**  log mm |
| --- | --- | --- | --- | --- | --- | --- | --- |
| *Acrostichum* | 10.15468/dl.z20xhn | 3.17 ± 0.19 | 2.41 ± 0.2 | 1.27 ± 0.6 | 64 ± 25 | 2.61 ± 0.25 | 2.17 ± 0.59 |
| *Adenanthera* | 10.15468/dl.qka228 | 3.26 ± 0.19 | 2.53 ± 0.16 | 1.24 ± 0.77 | 73 ± 34 | 2.75 ± 0.2 | 2.02 ± 0.75 |
| *Amanoa* | 10.15468/dl.zsz8zp | 3.36 ± 0.15 | 2.54 ± 0.15 | 1.64 ± 0.54 | 50 ± 19 | 2.59 ± 0.23 | 2.65 ± 0.53 |
| *Anacolosa* | 10.15468/dl.u6v0m6 | 3.2 ± 0.27 | 2.48 ± 0.2 | 1.26 ± 0.78 | 73 ± 41 | 2.76 ± 0.19 | 1.95 ± 0.79 |
| *Anemia* | 10.15468/dl.fnt3vm | 3.16 ± 0.15 | 2.35 ± 0.15 | 1.47 ± 0.53 | 52 ± 28 | 2.62 ± 0.17 | 2.21 ± 0.41 |
| Apocynaceae | 10.15468/dl.f4ajem | 2.94 ± 0.3 | 2.12 ± 0.31 | 1.22 ± 0.62 | 52 ± 31 | 2.33 ± 0.42 | 2.05 ± 0.56 |
| Araceae | 10.15468/dl.gthhtb | 3 ± 0.2 | 2.11 ± 0.24 | 1.51 ± 0.44 | 36 ± 24 | 2.37 ± 0.35 | 2.29 ± 0.34 |
| *Araucaria*/*Agathis* | 10.15468/dl.q69bzn | 3.24 ± 0.18 | 2.37 ± 0.18 | 1.82 ± 0.33 | 38 ± 21 | 2.7 ± 0.24 | 2.51 ± 0.31 |
| Arecaceae | 10.15468/dl.kevers | 3.17 ± 0.3 | 2.4 ± 0.26 | 1.27 ± 0.72 | 63 ± 31 | 2.55 ± 0.42 | 2.17 ± 0.72 |
| Asteraceae | 10.15468/dl.ns0e93 | 2.9 ± 0.25 | 2.02 ± 0.24 | 1.43 ± 0.5 | 36 ± 24 | 2.26 ± 0.39 | 2.21 ± 0.35 |
| Blechnaceae | 10.15468/dl.sus7ms | 3.09 ± 0.19 | 2.21 ± 0.23 | 1.58 ± 0.49 | 39 ± 27 | 2.47 ± 0.31 | 2.34 ± 0.43 |
| Bombacoideae | 10.15468/dl.ga5vvq | 3.11 ± 0.27 | 2.38 ± 0.22 | 0.97 ± 0.76 | 76 ± 32 | 2.46 ± 0.37 | 1.91 ± 0.9 |
| Buxaceae | 10.15468/dl.6g525b | 2.97 ± 0.21 | 2.07 ± 0.25 | 1.52 ± 0.41 | 33 ± 23 | 2.35 ± 0.35 | 2.24 ± 0.33 |
| *Caesalpinia* | 10.15468/dl.9ywqzk | 3 ± 0.31 | 2.28 ± 0.28 | 0.97 ± 0.63 | 75 ± 27 | 2.48 ± 0.37 | 1.9 ± 0.62 |
| *Calamus* | 10.15468/dl.kevers | 3.36 ± 0.15 | 2.52 ± 0.13 | 1.78 ± 0.52 | 46 ± 29 | 2.81 ± 0.18 | 2.53 ± 0.47 |
| *Calystegia* | 10.15468/dl.6uuu9f | 2.9 ± 0.16 | 1.98 ± 0.16 | 1.52 ± 0.42 | 28 ± 19 | 2.24 ± 0.34 | 2.29 ± 0.21 |
| *Ceratopetalum* | 10.15468/dl.x5ptjz | 3.14 ± 0.17 | 2.28 ± 0.2 | 1.72 ± 0.23 | 39 ± 19 | 2.65 ± 0.25 | 2.38 ± 0.21 |
| Chloranthaceae | 10.15468/dl.4u3zyw | 3.24 ± 0.2 | 2.42 ± 0.18 | 1.64 ± 0.45 | 50 ± 23 | 2.74 ± 0.19 | 2.32 ± 0.45 |
| *Cleistopholis* | 10.15468/dl.3uvb5h | 3.23 ± 0.14 | 2.48 ± 0.16 | 1 ± 0.54 | 64 ± 16 | 2.57 ± 0.22 | 2.29 ± 0.68 |
| Clusiaceae | 10.15468/dl.v6xtf7 | 3.27 ± 0.19 | 2.47 ± 0.17 | 1.47 ± 0.6 | 56 ± 25 | 2.63 ± 0.25 | 2.38 ± 0.6 |
| *Croton* | 10.15468/dl.q8fdmv | 3.03 ± 0.3 | 2.27 ± 0.28 | 1.08 ± 0.67 | 67 ± 29 | 2.46 ± 0.39 | 1.97 ± 0.65 |
| *Crudia* | 10.15468/dl.dgg4vi | 3.37 ± 0.14 | 2.56 ± 0.14 | 1.58 ± 0.62 | 52 ± 25 | 2.62 ± 0.24 | 2.61 ± 0.57 |
| *Ctenolophon* | 10.15468/dl.xb2gwf | 3.46 ± 0.11 | 2.57 ± 0.13 | 1.89 ± 0.71 | 33 ± 18 | 2.84 ± 0.11 | 2.66 ± 0.64 |
| Cupanieae | 10.15468/dl.kwwwpc | 3.22 ± 0.18 | 2.43 ± 0.17 | 1.41 ± 0.58 | 58 ± 25 | 2.61 ± 0.22 | 2.28 ± 0.57 |
| Cupressaceae | 10.15468/dl.hcstys | 2.88 ± 0.23 | 2.01 ± 0.24 | 1.41 ± 0.47 | 37 ± 24 | 2.26 ± 0.36 | 2.18 ± 0.37 |
| Cyatheaceae | 10.15468/dl.rnaahg | 3.24 ± 0.2 | 2.39 ± 0.21 | 1.69 ± 0.44 | 44 ± 25 | 2.66 ± 0.24 | 2.43 ± 0.43 |
| *Cynometra* | 10.15468/dl.z3js1z | 3.25 ± 0.18 | 2.49 ± 0.16 | 1.33 ± 0.62 | 64 ± 26 | 2.64 ± 0.27 | 2.3 ± 0.64 |
| Cyperaceae | 10.15468/dl.ll6mya | 2.93 ± 0.22 | 2.04 ± 0.23 | 1.48 ± 0.48 | 34 ± 23 | 2.31 ± 0.33 | 2.25 ± 0.37 |
| Fabaceae | 10.15468/dl.3f0xgi | 2.9 ± 0.28 | 2.07 ± 0.29 | 1.27 ± 0.58 | 46 ± 30 | 2.27 ± 0.41 | 2.12 ± 0.49 |
| *Gevuina*+*Hicksbeachia* | 10.15468/dl.j4lyef | 3.22 ± 0.13 | 2.45 ± 0.15 | 1.6 ± 0.21 | 62 ± 18 | 2.69 ± 0.34 | 2.4 ± 0.32 |
| Gleicheniaceae | 10.15468/dl.xtwwir | 3.2 ± 0.21 | 2.37 ± 0.22 | 1.57 ± 0.55 | 49 ± 28 | 2.64 ± 0.26 | 2.31 ± 0.54 |
| *Gunnera* | 10.15468/dl.87dbkb | 3.16 ± 0.25 | 2.26 ± 0.24 | 1.73 ± 0.45 | 34 ± 23 | 2.55 ± 0.3 | 2.43 ± 0.45 |
| *Illicium* | 10.15468/dl.va89fd | 3.21 ± 0.14 | 2.4 ± 0.16 | 1.63 ± 0.34 | 51 ± 22 | 2.78 ± 0.16 | 2.24 ± 0.34 |
| Iridaceae | 10.15468/dl.2uxusc | 2.91 ± 0.23 | 2.04 ± 0.23 | 1.41 ± 0.47 | 39 ± 24 | 2.27 ± 0.4 | 2.2 ± 0.36 |
| Lecythidaceae | 10.15468/dl.shmt62 | 3.27 ± 0.19 | 2.49 ± 0.16 | 1.36 ± 0.69 | 61 ± 28 | 2.61 ± 0.26 | 2.32 ± 0.71 |
| Lepidocaryeae | 10.15468/dl.kevers | 3.26 ± 0.18 | 2.48 ± 0.14 | 1.15 ± 0.75 | 62 ± 25 | 2.53 ± 0.28 | 2.41 ± 0.73 |
| Liliaceae | 10.15468/dl.yhq44l | 2.9 ± 0.2 | 2.02 ± 0.21 | 1.42 ± 0.48 | 36 ± 24 | 2.23 ± 0.42 | 2.24 ± 0.3 |
| *Ludwigia* | 10.15468/dl.tnxfi9 | 3.05 ± 0.26 | 2.25 ± 0.27 | 1.25 ± 0.66 | 56 ± 34 | 2.44 ± 0.38 | 2.11 ± 0.62 |
| *Lygodium* | 10.15468/dl.fia0r5 | 3.22 ± 0.17 | 2.43 ± 0.18 | 1.41 ± 0.64 | 59 ± 30 | 2.66 ± 0.22 | 2.21 ± 0.6 |
| Matoniaceae | 10.15468/dl.pxa8e2 | 3.48 ± 0.09 | 2.6 ± 0.13 | 2.18 ± 0.13 | 31 ± 14 | 2.82 ± 0.1 | 2.97 ± 0.15 |
| *Monstera*/*Gonatopus* | 10.15468/dl.rhpbd9 | 3.27 ± 0.21 | 2.46 ± 0.19 | 1.53 ± 0.52 | 56 ± 23 | 2.61 ± 0.26 | 2.44 ± 0.48 |
| Nypoideae | 10.15468/dl.kevers | 3.29 ± 0.18 | 2.55 ± 0.13 | 1.16 ± 0.77 | 76 ± 29 | 2.77 ± 0.18 | 1.89 ± 0.76 |
| Osmundaceae | 10.15468/dl.8xsj6w | 3.04 ± 0.19 | 2.12 ± 0.21 | 1.66 ± 0.36 | 29 ± 19 | 2.43 ± 0.28 | 2.36 ± 0.28 |
| Poaceae | 10.15468/dl.xnfteg | 2.89 ± 0.24 | 2.01 ± 0.23 | 1.43 ± 0.49 | 35 ± 23 | 2.26 ± 0.36 | 2.21 ± 0.35 |
| Podocarpaceae | 10.15468/dl.98m3pn | 3.18 ± 0.2 | 2.32 ± 0.22 | 1.65 ± 0.43 | 43 ± 26 | 2.61 ± 0.28 | 2.38 ± 0.42 |
| Polygalaceae | 10.15468/dl.v4j7yx | 2.96 ± 0.2 | 2.08 ± 0.23 | 1.46 ± 0.46 | 38 ± 25 | 2.34 ± 0.32 | 2.24 ± 0.37 |
| Polygonaceae | 10.15468/dl.1yaddr | 2.89 ± 0.23 | 2 ± 0.23 | 1.46 ± 0.46 | 34 ± 22 | 2.27 ± 0.36 | 2.22 ± 0.3 |
| Polypodiaceae | 10.15468/dl.gesnzu | 3.05 ± 0.23 | 2.18 ± 0.26 | 1.54 ± 0.46 | 39 ± 25 | 2.45 ± 0.32 | 2.29 ± 0.4 |
| Proteaceae | 10.15468/dl.z7gsmv | 2.81 ± 0.35 | 2.03 ± 0.38 | 1.04 ± 0.59 | 58 ± 32 | 2.27 ± 0.39 | 1.86 ± 0.56 |
| *Pteris* | 10.15468/dl.kkbbu1 | 3.16 ± 0.23 | 2.35 ± 0.23 | 1.47 ± 0.52 | 54 ± 26 | 2.61 ± 0.33 | 2.24 ± 0.5 |
| Rhizophoraceae | 10.15468/dl.esadj3 | 3.21 ± 0.27 | 2.45 ± 0.23 | 1.29 ± 0.73 | 64 ± 30 | 2.62 ± 0.29 | 2.2 ± 0.71 |
| Sapotaceae | 10.15468/dl.hnyoqw | 3.16 ± 0.23 | 2.38 ± 0.21 | 1.24 ± 0.68 | 63 ± 28 | 2.56 ± 0.28 | 2.15 ± 0.67 |
| Schizaeaceae | 10.15468/dl.q9jcj5 | 3.21 ± 0.22 | 2.36 ± 0.23 | 1.63 ± 0.59 | 44 ± 29 | 2.61 ± 0.29 | 2.37 ± 0.58 |
| Simaroubaceae | 10.15468/dl.ce4ydt | 3.15 ± 0.28 | 2.38 ± 0.25 | 1.25 ± 0.66 | 62 ± 26 | 2.55 ± 0.32 | 2.2 ± 0.61 |
| *Stenochlaena* | 10.15468/dl.5m8y5m | 3.3 ± 0.19 | 2.53 ± 0.11 | 1.26 ± 0.95 | 66 ± 38 | 2.74 ± 0.19 | 2.04 ± 0.88 |
| Sterculioideae | 10.15468/dl.zhwcud | 3.05 ± 0.29 | 2.3 ± 0.3 | 1.03 ± 0.7 | 68 ± 33 | 2.49 ± 0.31 | 1.93 ± 0.73 |
| *Symphonia* | 10.15468/dl.6dvk87 | 3.33 ± 0.17 | 2.52 ± 0.15 | 1.57 ± 0.54 | 54 ± 21 | 2.69 ± 0.26 | 2.52 ± 0.51 |
| Ulmaceae | 10.15468/dl.sevcne | 2.92 ± 0.16 | 2 ± 0.17 | 1.58 ± 0.32 | 27 ± 16 | 2.33 ± 0.24 | 2.25 ± 0.24 |

**Table S2**. Geodetic coordinates of occurrences from the Global Biodiversity Information Facility (GBIF)
